# Human cytomegalovirus RNA2.7 inhibits ferroptosis by upregulating ferritin and GSH via promoting ZNF395 degradation

**DOI:** 10.1371/journal.ppat.1012815

**Published:** 2024-12-26

**Authors:** Mingyi Xu, Shan Ruan, Jingxuan Sun, Jianming Li, Dan Chen, Yanping Ma, Ying Qi, Zhongyang Liu, Qiang Ruan, Yujing Huang

**Affiliations:** 1 Virology Laboratory, Shengjing Hospital of China Medical University, Shenyang, Liaoning, China; 2 Department of Gerontology and Geriatrics, Shengjing Hospital of China Medical University, Shenyang, Liaoning, China; 3 Department of Gynaecology, Liaoning Cancer Hospital & Institute, Shenyang, China; 4 Department of Pediatrics, Shengjing Hospital of China Medical University, Shenyang, Liaoning, China; 5 Department of Obstetrics and Gynecocology, Shengjing Hospital of China Medical University, Shenyang, Liaoning, China; Leibniz Institute of Virology (LIV), GERMANY

## Abstract

Human cytomegalovirus (HCMV) is a herpes virus with a long replication cycle. HCMV encoded long non-coding RNA termed RNA2.7 is the dominant transcript with a length of about 2.5kb, accounting for 25% of total viral transcripts. Studies have shown that HCMV RNA2.7 inhibits apoptosis caused by infection. The effect of RNA2.7 on other forms of cell death is still unclear. In this work, we found that RNA2.7 deletion significantly decreased the viability of HCMV-infected cells, while treatment with ferroptosis inhibitor Fer-1 rescued the infection-induced cell death, demonstrating an anti-ferroptosis role of RNA2.7. The results further showed that RNA2.7 inhibited ferroptosis via enhancing Ferritin Heavy Chain 1 (FTH1) and Solute Carrier Family 7 Member 11 (SLC7A11) expression in Erastin treated cells without involving other viral components. Pooled Genome-wide CRISPR screening revealed zinc finger protein 395 (ZNF395) as a new regulator repressing the expression of FTH1 and SLC7A11. HCMV RNA2.7 promoted proteasome-mediated degradation of ZNF395 that resulted in upregulation of FTH1 and SLC7A11 to inhibit ferroptosis, therefore maintain survival in host cells and complete replication of virus.

## Author summary

Human cytomegalovirus (HCMV) exhibits a latency that persists throughout life following the primary infection. Approximately 0.6% of newborns are HCMV-congenitally infected, 20–25% of which are symptomatic at birth or will develop long-term sequelae with a substantial public health impact. HCMV is slow-replicating virus that maintains the survival of the host cell to ensure virus replication. Several HCMV encoded proteins have been found to inhibit apoptosis and necroptosis following viral infection. However, the role of HCMV in other types of programmed cell death such as ferroptosis has not been clarified. In this study, we found that HCMV RNA2.7 inhibited ferroptosis independent on other viral components. Then we tried to explore how RNA2.7 inhibit ferroptosis and found RNA2.7 effectively increased ferritin and GSH contents to dampen ferroptosis. However, the regulation of RNA2.7 on ferritin and GSH contents was not depend on any previously reported regulators. Therefore, a pooled genome-wide CRISPR was employed and ZNF395 was determined as a newly regulator that promoted ferroptosis. Then, we confirmed that HCMV RNA2.7 promoted proteasome-mediated degradation of ZNF395 that resulted in upregulation of FTH1 and SLC7A11 to inhibit ferroptosis, therefore maintain survival in host cells and complete replication of virus.

## Introduction

Human cytomegalovirus (HCMV) is the largest known herpes virus, exhibiting a latency that persists throughout life following the primary infection [1–3]. Despite being usually asymptomatic in adults, reactivation from latency is possible in certain immunocompromised circumstances, such as acquired immunodeficiency syndrome and organ or stem cell transplantations, increasing the burden of morbidity or mortality in the affected populations [4]. Moreover, approximately 0.6% of newborns are HCMV-congenitally infected and, among these, 20–25% are symptomatic at birth or will develop long-term sequelae, with a substantial public health impact [5–7]. The HCMV infection has been associated with the development of some autoimmune diseases and degenerative disorders, by inducing chronic inflammatory response [8]. However, the mechanism by which HCMV infection causes diseases is still unclear.

Viruses are ubiquitous intracellular genetic parasites that heavily rely on the infected cell to complete their replication life cycle. Due to this dependency, the viruses modulate cellular processes in the host cell including cell survival and cell death [9]. Alteration to the programmed cell death (PCD) in the infected hosts may have direct and indirect effects on viral pathogenesis and antiviral immunity [10]. HCMV are slow-replicating viruses that maintains the survival of the host cell to ensure virus replication [11]. Several HCMV encoded proteins have been found to inhibit apoptosis and necroptosis following viral infection. It has been reported that HCMV encodes UL37 exon 1 protein (UL37x1) to inhibit intrinsic apoptosis by binding to pro-apoptotic Bax and prevent mitochondrial cytochrome *c* release into cytosols. On the other hand, HCMV-encoded UL36, probably better known for its other name, the viral inhibitor of caspase-8 activation (vICA), has been found to inhibit extrinsic apoptosis by directly binding to pro–caspase-8, thereby inhibiting its cleavage into active caspase-8. Additionally, UL36 has also been reported to promote proteasomal degradation of mixed lineage kinase domain-like protein (MLKL) in infected cells as well, therefore inhibit necroptosis. HCMV coding protein has both extraordinary capacities to interfere with host immune defense and inhibition of PCD, while the other forms of PCDs inhibited by HCMV are still relatively understudied, such as ferroptosis.

Ferroptosis is a newly defined form of programmed cell death characterized by the accumulation of iron-dependent lipid peroxides [12]. The dependence of ferroptosis on Fe^2+^ loads distinguish it from other types of regulated cell deaths [13]. Excessive accumulation of Fe^2+^ catalyzes the formation of peroxides leading to cell death [14]. The survival of viruses is maintained by various iron-dependent cellular processes. Therefore, HCMV can regulate iron metabolism. For instance, intracellular iron levels were associated with cytomegaly, and the viral protein US2 degrades homeostatic iron regulator (HFE) to disrupt iron uptake. Furthermore, pUL38 of HCMV can protect cells from ferritinophagy by stabilizing the Nuclear Receptor Coactivator 4 (NCOA4) and preventing ferritin heavy chain from degradation. It has been reported that HCMV upregulates membrane associated RING CH E3 ligase 1 (MRCH1) to increase TfR and iron accumulation early following infection. However, little is known on how HCMV survives in an environment with excessive iron levels. To prevent the detrimental effects of excessive peroxides, cells have evolved inherent pathways and reductase system for protection. Emerging evidence has shown that pathogenic infections contribute to ferroptosis [15], especially viral infections. It has been reported that Coxsackievirus A6 promotes the dissemination of viral particles by inducing ferroptosis in host cells [16,17]. The Epstein-Barr virus (EBV) latent gene Epstein–Barr nuclear antigen 1 (EBNA1) can prevent ferroptosis via activating the Keap1-NRF2 signaling pathway to upregulate the expression of solute carrier family 7 member 11 (SLC7A11) and glutathione peroxidase 4 (GPX4) thereby promoting chemoresistance and tumor progression in nasopharyngeal carcinoma [18,19]. However, the relationship between HCMV infection and ferroptosis need to be further studied.

HCMV encoded long non-coding RNA termed RNA2.7 is the dominant transcript with a length of about 2.5kb, accounting for 25% of total viral transcripts [20]. In previous studies, RNA2.7 was not found to be essential to viral replication under normal culture conditions. However, in recent years, several investigations have confirmed that RNA2.7 may affect viral replication under special culture conditions. RNA2.7 binds to GRIM-19 and stabilizes the mitochondrial membrane potential, helping the host cell to overcome glucose-depleted condition and complete the virus’s life cycle [21]. Moreover, RNA2.7 can protect CD14+ monocytes against reactive oxygen species (ROS)-induced apoptosis via increasing superoxide dismutase 2 (SOD2) and supporting latent HCMV infection [22]. Currently, most studies on HCMV RNA2.7 function have focused on its inhibitory effect on apoptosis. Thus, little is known about the role of RNA2.7 in other forms of PCDs besides apoptosis.

This study demonstrates that RNA2.7 is essential for HCMV-infected cell survival. By generating an HCMV mutant lacking RNA2.7, we observed a significant decrease in cell viability. Importantly, this infection-induced cell death was rescued by the ferroptosis inhibitor Fer-1, suggesting that RNA2.7 protects cells from ferroptosis. The results further demonstrated that HCMV RNA2.7 inhibited ferroptosis via enhancing ferritin heavy chain 1 (FTH1) and SLC7A11 expression levels in Erastin-treated cells without involving viral components. The pooled genome-wide CRISPR screen and validation experiments confirmed that zinc finger protein 395 (ZNF395) suppressed the expression of FTH1 and SLC7A11. HCMV RNA2.7 enhanced the ubiquitylation of ZNF395 and promoted the proteasome-mediated degradation of ZNF395, which resulted in upregulation of the expression of FTH1 and SLC7A11 to inhibit ferroptosis.

## Results

### RNA2.7 inhibits ferroptosis in HCMV infected cells

**Fig 1:**
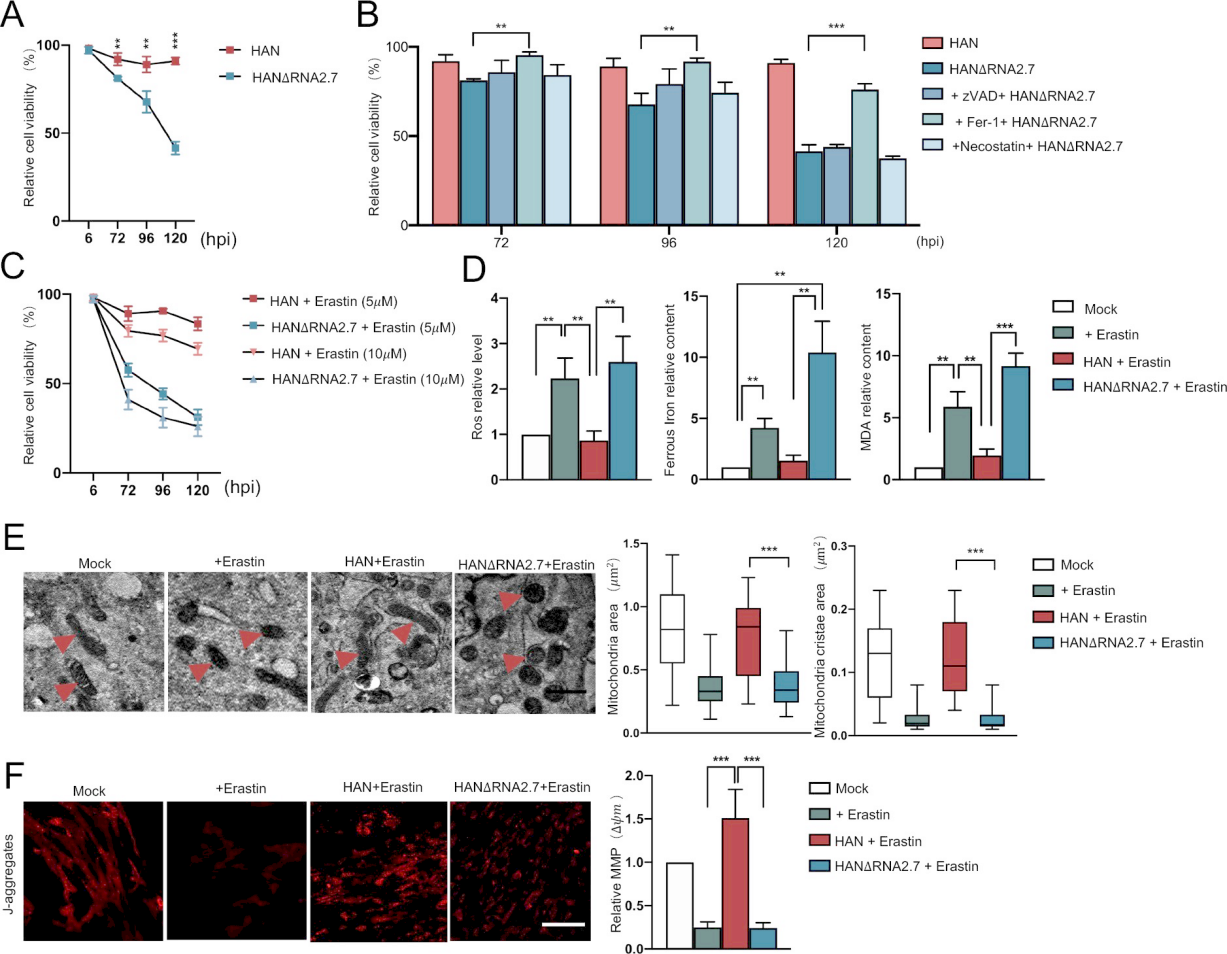
RNA2.7 inhibits ferroptosis in HCMV-infected cells. (A) HELF cells were infected with HAN or HANΔRNA2.7 (MOI = 5). Cell viabilities were evaluated using cell viability assay at 6, 72, 96 and 120 hours post-infection (hpi), respectively. Relative viabilities were calculated using uninfected HELF cells as control. All experiments were conducted independently three times. Error bars represent the mean ± SD from three independent experiments. (B) HELF cells were infected with HANΔRNA2.7 (MOI = 5). At 24 hpi, z-VAD (10*μ*M), Fer-1 (5*m*M), and Necostatin (10*μ*M) were added into the supernatants of infected cells. Cell viabilities were evaluated using cell viability assay at 72, 96 and 120 hours post-treatment. Relative viabilities were calculated using uninfected cells as control. All experiments were conducted independently three times. Error bars represent the mean ± SD from three independent experiments. (C) HELF cells were infected with either HAN or HANΔRNA2.7 at an MOI of 1 At 24 hpi, Erastin was added into the supernatants at a final concentration of 5*μ*M or 10*μ*M. Cell viabilities were evaluated using cell viability assay at 6, 72, 96 and 120 hpi. Relative viabilities were calculated according to uninfected HELF cells. All experiments were conducted independently three times. Error bars represent the mean ± SD from three independent experiments. (D) HELF cells were infected with HAN or HANΔRNA2.7 (MOI = 1). Erastin was added into the supernatant at 24 hpi with a final concentration of 5*μ*M. Contents of reactive oxygen species (ROS), ferrous iron, and malondialdehyde (MDA) were measured using ROS assay, MDA assay and iron assay at 24 hours post treatment, respectively. Relative contents were calculated using mock group as control. All experiments were conducted independently three times. Error bars represent the mean ± SD from three independent experiments. (E) HELF cells were infected with HAN or HANΔRNA2.7 (MOI = 1) and followed by Erastin treatment (5*μ*M) at 24 hpi. Cells were fixed at 24 hours post treatment. Mitochondrial were observed and measured via transmission electron microscopy. Left: representative images are shown; red triangles indicate mitochondria exhibiting distinct structures; scale bars = 500*n*m. Right: mitochondrial area and cristae area were quantified using ImageJ across four groups of cells, measuring a total of 30 mitochondria in each group. Statistical analyses were conducted using GraphPad Prism version 5.0, calculating the maximum, minimum, median, upper quartile, and lower quartile for both mitochondrial area and cristae area. Error bars represent mean ± SD. (F) HELF cells were infected with HAN or HANΔRNA2.7 (MOI = 1) and followed by Erastin treatment (5*μ*M) at 24 hpi. Cells were stained with JC-1 probes at 24 hours post treatment. Images were obtained and analyzed via fluorescence confocal microscopy. The magnification of taken pictures was 300x. Left: representative images are shown; scale bars = 100*μ*m; Right: fluorescence intensities indicating mitochondrial membrane potential (MMP) were measured utilizing ImageJ software. Relative intensities were calculated according to the mock group as control. Error bars denote mean ± SD. **, *P* < 0.01; ***, *P* < 0.001.

To investigate the effect of HCMV RNA2.7 on cell survival following infection, we analyzed the cell viability between cells infected with HCMV clinical strain HAN and RNA2.7-deleted mutant (HANΔRNA2.7) at a multiplicity of infection (MOI) of 5. It was observed that the viability of the cells infected with HAN was approximately 98.17 ± 3.57% at 7 days post infection (dpi). However, in the absence of RNA2.7, the viability of HANΔRNA2.7 infected cells decreased to 81.1 ± 0.96% at 3 dpi, and further dropped to 41.50 ± 3.63% at 7 dpi (Fig 1A). This indicated that HCMV RNA2.7 might protect infected from cell death induced by infection.

To determine the major PCD pattern prevented by RNA2.7, various inhibitors targeting different forms of PCDs were added into HANΔRNA2.7 infected cells, including Fer-1 for ferroptosis, necrostatin-1 for necroptosis and z-VAD for pan-caspase-mediated apoptosis [23]. Subsequently, the cell viabilities were evaluated and compared between treatments. The results showed that inhibition of ferroptosis by Fer-1 effectively prevented cell death caused by HANΔRNA2.7 infection (Fig 1B). Fer-1 treatment significantly enhanced cell viability following HANΔRNA2.7 infection, maintaining approximately 76.02 ± 3.29% viability even seven days post-infection. This is in contrast to the marked decrease in viability observed in the absence of Fer-1. No difference was found for intracellular levels of cleaved caspase-3 and cytochrome C between HCMV-infected cells with or without RNA2.7, which indicated that the HCMV infection-induced cell death did not increase cell leak contents and was distinct from caspase-dependent apoptosis (S1A Fig). Moreover, the cell death could be effectively rescued by adding iron chelator deferoxamine (DFO) and ROS scavenger N-Acetylcysteine (NAC) into HANΔRNA2.7 infected cells (S1B Fig). These findings suggested that RNA2.7 might function to counteract HCMV infection-induced ferroptosis and promote cell survival.

Erastin has been extensively applied to induce ferroptosis *in vivo* owing to its ability to increase intracellular Fe^2+^ content and inhibit the activity of the cellular reductase system. To further confirm the anti-ferroptosis effect of HCMV RNA2.7, HELF cells were infected with HAN or HANΔRNA2.7 and then treated with Erastin (5*μ*M & 10*μ*M). HANΔRNA2.7-infected cells exhibited a progressive decline in viability over time, irrespective of Erastin concentration. At 7 dpi, viability was reduced to 31.14 ± 4.33% (5*μ*M Erastin) and 26.04 ± 5.46% (10*μ*M Erastin). In contrast, HAN-infected cells maintained higher viability with increasing time and Erastin exposure, reaching 69.45 ± 3.33% at 7 dpi with 10*μ*M Erastin (Fig 1C). It was further confirmed that knock down of RNA2.7 in HCMV strain TB40/E also resulted in reduced viabilities in infected cells post Erastin treatment (S1C–S1D Fig). These results indicated that RNA2.7 expression in HCMV-infected cells confered resistance to Erastin-induced ferroptosis.

Ferroptosis is mainly characterized by excessive intracellular accumulation of iron and redox imbalance [24,25]. In condition of Erastin/RSL3 treatment, the intracellular levels of Fe^2+^, ROS, and malondialdehyde (MDA) were significantly increased to 4.23±0.76, 6.17±1.81 and 2.79±0.51, indicating the occurrence of ferroptosis (Figs 1D and S1E). Specifically, the ferroptosis hallmarks presented significant changes in HANΔRNA2.7 infected cells similar to those in Erastin/RSL3 treated cells. HANΔRNA2.7 infection increased the Fe^2+^ level to 10.4 ± 2.27, the ROS level to 2.60 ± 0.19, and the MDA level to 9.16 ± 1.38 relative to the mock-infected cells. However, the intracellular levels of Fe^2+^, ROS and MDA in HAN-infected cells with RNA2.7 were almost comparable to levels in HELF cells.

Ferroptosis has been shown to cause mitochondrial dysfunction [14]. In HANΔRNA2.7-infected cells, the mitochondria appeared shrunken and mitochondrial membrane density was significantly reduced (Fig 1E). The number of mitochondria and mitochondrial cristae areas were reduced to 0.38 and 0.028*μ*m^2^, respectively post-infection and Erastin treatment. Mitochondrial membrane potential (MMP, Δψm) is commonly used as an indicator of mitochondrial function. Compared to HAN-infected cells, HANΔRNA2.7-infected cells exhibited a significant loss of MMP from 1.49 ± 0.63 to 0.75 ± 0.06, indicative of mitochondrial dysfunction associated with ferroptosis (Figs 1F and S1F). In contrast, HAN-infected cells maintained normal mitochondrial size and distinct inner cristae structure even following Erastin treatment, suggesting that RNA2.7 preserves mitochondrial integrity. These results suggested that HCMV RNA2.7 rescued mitochondrial function in infected cells following Erastin treatment.

### HCMV RNA2.7 inhibits ferroptosis independent on other viral components

**Fig 2:**
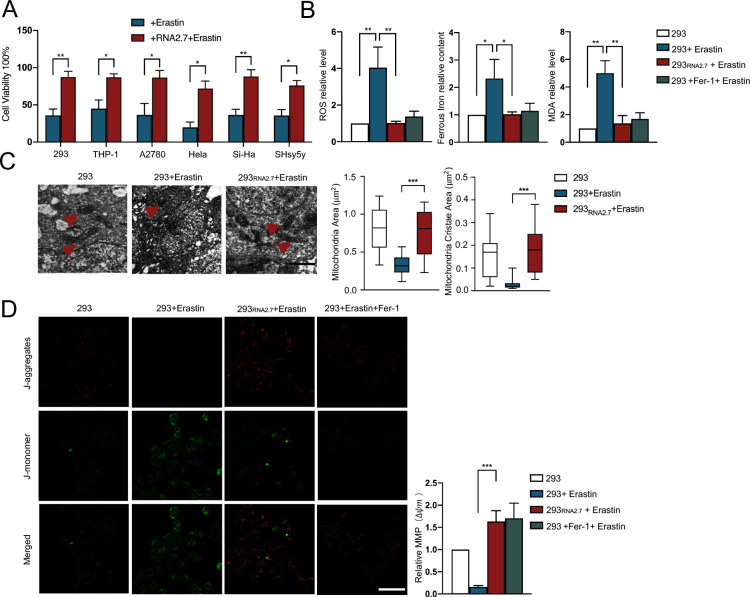
HCMV RNA2.7 inhibits ferroptosis independent on other viral components. (A) 293, THP-1, A2780, HeLa, Si-Ha and SHsy5y cells were transfected with vectors expressing RNA2.7 and subsequently treated with Erastin (10*μ*M) at 24 hours post-transfection. Cell viabilities were evaluated using viability assay at 24 hours post treatment. Relative viabilities were calculated using normal cells as control. All experiments were conducted independently three times. Error bars represent the mean ± SD from three independent experiments. (B) 293 cells stably expressing RNA2.7 were constructed (293_RNA2.7_). Fer-1 (10*μ*M) was pretreated in 293 cells 2 hours before Erastin treatment and served as a positive anti-ferroptotic control. 293, 293_RNA2.7_ and Fer-1 pretreated 293 cells were treated with Erastin (5*μ*M). Intracellular levels of ROS, ferrous iron, and MDA were measured respectively at 24 hours post treatment. Relative levels of ferroptotic hallmarks were calculated using untreated 293 cells as control. Error bars represent the mean ± SD from three independent experiments. (C) Erastin (5*μ*M) was added to both 293 and 293_RNA2.7_ cells. Cells were fixed at 24 hours post treatment. Mitochondrial were observed and measured via transmission electron microscopy. Left: representative images are shown; red triangles indicate mitochondria exhibiting distinct structures; scale bars = 500*n*m. Right: mitochondrial area and cristae area were quantified using ImageJ across four groups of cells, measuring a total of 30 mitochondria in each group. Statistical analyses were conducted using GraphPad Prism version 5.0, calculating the maximum, minimum, median, upper quartile, and lower quartile for both mitochondrial area and cristae area. Error bars represent mean ± SD. (D) 293 and 293_RNA2.7_ cells were treated with Erastin (5*μ*M). Cells were stained with JC-1 probes at 24 hours post treatment. Images were obtained and analyzed via fluorescence confocal microscopy. The magnification of taken pictures was 300x. Left: representative images are shown; scale bars = 100*μ*m; Right: fluorescence intensities indicating mitochondrial membrane potential (MMP) were measured utilizing ImageJ software. Relative intensities were calculated according to the mock group as control. Error bars denote mean ± SD. *, *P* < 0.05; **, *P* < 0.01; ***, *P* < 0.001.

To elucidate the anti-ferroptotic role of RNA2.7, expression vectors encoding HCMV RNA2.7 were generated and transfected into a panel of cell lines, including 293, THP-1, A2780, HeLa, SiHa and SHsy5y. The viabilities of cells were evaluated and compared post-Erastin treatment. Compared to normal cells, the viabilities of cells treated with Erastin decreased from 45.04 ± 11.55% to 19.88 ± 7.08%, while that of cells expressing RNA2.7 was maintained at 71.92 ± 10.02% (Fig 2A). The inhibition of ferroptosis by RNA2.7 was further confirmed by staining of dead cells with Erastin (S2 Fig). Collectively, these results demonstrated that HCMV RNA2.7 inhibited Erastin-induced ferroptosis in various cell lines.

To determine the inhibitory effect of RNA2.7 on ferroptosis, a 293 cell line stably expressing RNA2.7 (293_RNA2.7_) was constructed. The 293 cells and 293_RNA2.7_ cells were treated with Erastin followed by measurement of intracellular levels of ROS, Fe^2+^ and MDA in different groups (Fig 2B). In addition, 293 cells pretreated with Fer-1 were introduced as a positive control with anti-ferroptotic effect. Compared to normal 293 cells, cells treated with Erastin exhibited increased levels of ROS and Fe^2+^ levels to 2.33 ± 0.67 and 4.04 ± 1.13, respectively. Erastin treatment induced ferroptosis by elevating ROS and Fe^2+^ levels, leading to a 5.00 ± 0.90-fold increase in MDA production. In contrast, RNA2.7-expressing cells exhibited no significant alterations in ROS, Fe^2+^ or MDA levels upon Erastin challenge. The increases of ferroptosis hallmarks by Erastin induction were eliminated by Fer-1 pre-treatment. Furthermore, RNA2.7 mitigated Erastin-induced mitochondrial damage, as evidenced by the preservation of mitochondrial morphology and membrane potential (Fig 2C–2D). RNA2.7 was also confirmed to inhibit ferroptosis induced by two more ferroptosis inducers (ML162 and RSL3) (S3 Fig). These results confirmed that HCMV RNA2.7 inhibited Erastin-induced ferroptosis independent on other viral components.

### HCMV RNA2.7 improves intracellular iron storage and increases the reductase activity of GPX4

**Fig 3:**
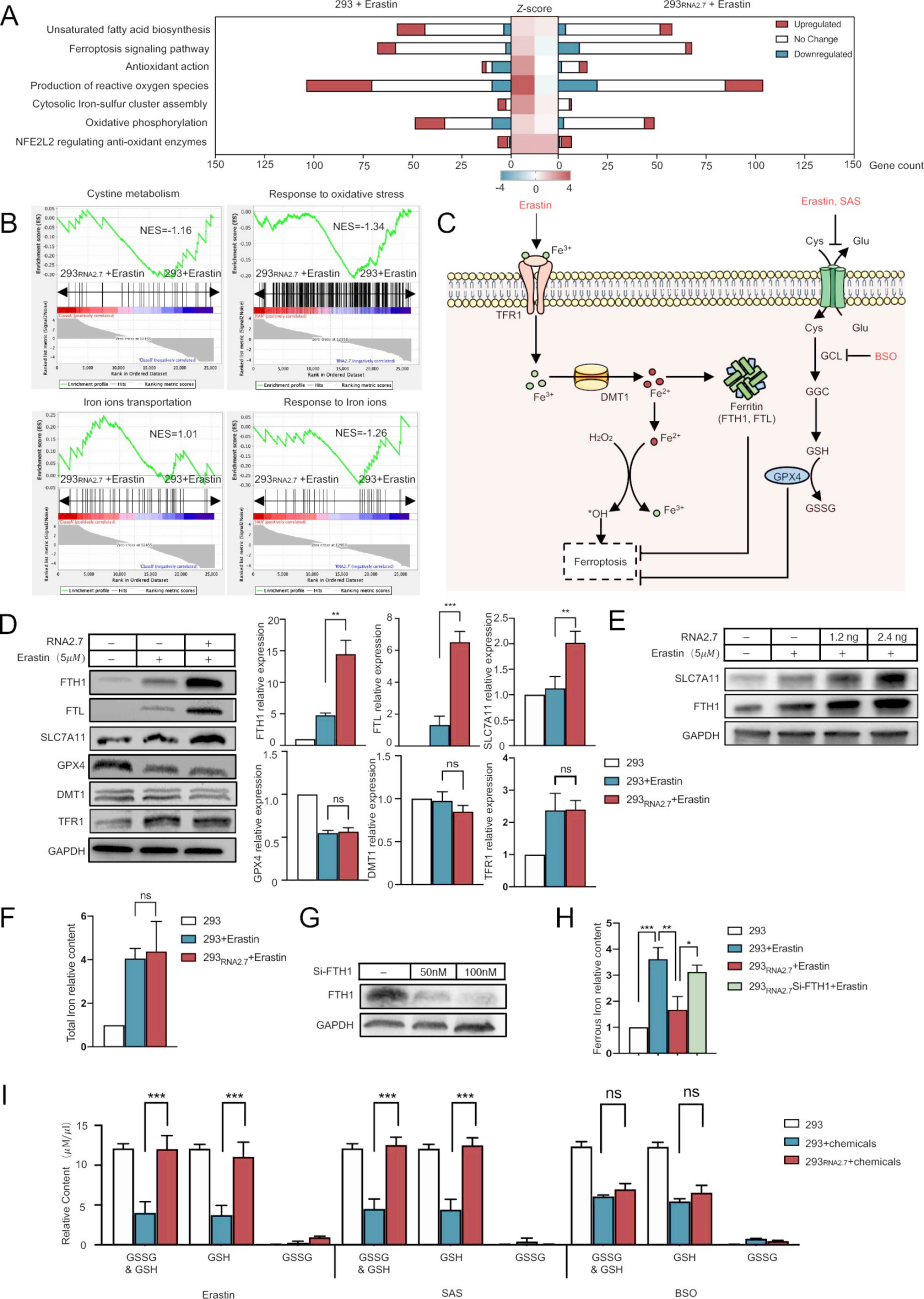
HCMV RNA2.7 improves intracellular iron storage and increases the reductase activity of GPX4. (A) 293 and 293_RNA2.7_ cells were treated with Erastin (5*μ*M) or not. Total RNA was extracted at 24 hours post treatment and sequenced by Illumina Novaseq platform. Transcriptomic profiles were analyzed using IPA software. Bars in red indicate gene counts of upregulated genes; bars in blue indicate gene counts of downregulated genes; bars in white indicate gene counts of genes exhibiting no significant change. *Z*-scores for each pathway in both 293 and 293_RNA2.7_ cells are displayed in the central heatmap. (B) NES scores of pathways including cysteine metabolism, response to oxidative stress, iron ion transportation, and response to iron ions were calculated using software GSEA. (C) A schematic abstract presents the major targets of Erastin, Sulfasalazine, and BSO in ferroptosis. (D) 293 and 293_RNA2.7_ cells were treated with Erastin (5*μ*M) and total cellular proteins were extracted at 24 hours post treatment. The expressions of ferroptosis-related genes, including FTH1, FTL, SLC7A11, GPX4, DMT1, and TFR1, were assessed by Western blots. GAPDH served as a loading control. Grey values were quantified using ImageJ software and the relative grey values were calculated using untreated group as control. Error bars represent mean ± SD from three independent experiments. (E) 293 cells were transfected with 1.2*n*g and 2.4*n*g vectors expressing RNA2.7, respectively. At 24 hours post-transfection, cells were treated with Erastin (5*μ*M). The protein levels of FTH1 and SLC7A11 were assessed by Western blots at 24 hours post treatment. (F) 293 and 293_RNA2.7_ cells were treated with Erastin (5*μ*M). Cells were lysed at 24 hours following treatment, and total iron contents in the cells were evaluated using Iron Assay. Relative total iron levels were calculated using untreated cells as control. Error bars represent mean ± SD from three independent experiments. (G) The 293_RNA2.7_ cells were transfected with siRNAs targeting FTH1 (Si-FTH1) at final concentrations of 50*n*M and 100*n*M, respectively. The knockdown efficiency of FTH1 was confirmed via Western blots. GAPDH served as a loading control. (H) 293_RNA2.7_ cells were transfected with Si-FTH1 at a final concentration of 100*n*M, and then treated with Erastin (5*μ*M) at 24 hours post transfection. Fe^2+^ levels were quantified using Iron Assay at 24 hours post Erastin treatment; relative Fe^2+^ contents were calculated using untreated group as control. (I) 293 and 293_RNA2.7_ cells were treated with Erastin (5*μ*M), SAS (5*μ*M), or BSO (10*μ*M). Cells were harvested at 24 hours post treatment. GSH and GSSG contents within these samples were measured utilizing GSH & GSSG assay while relative quantities were calculated according to the protein concentrations ascertained through BCA assay. Error bars indicate mean ± SD derived from three independent experimental replicates. *, *P* <0 .05; **, *P* <0 .01; ***, *P* <0 .001; ns, no significance.

To investigate the mechanism by which RNA2.7 inhibited ferroptosis, the transcriptomic profiles were analyzed and compared between 293 and 293_RNA2.7_ cells after Erastin treatment. The activities of various pathways associated with different genes were determined by *z*-scores. In the absence of HCMV RNA2.7, the Erastin treatment significantly increased the production of ROS and activation of the ferroptosis signaling pathway with *z*-scores of 3.6 and 1.9, respectively (Fig 3A). Similarly, pathways associated with ferroptosis, such as antioxidant action and cytosolic Iron-sulfur cluster assembly pathways, were also activated in Erastin-treated 293 cells with *z*-scores more than 2.0. The activation of gene transcriptions in these ferroptosis-associated pathways were later confirmed using quantitative PCR assay (S4A Fig). In cells expressing RNA2.7, no activation was observed in pathways described above with z-scores less than 0.5. Nuclear factor erythroid 2 related factor 2 (NFE2L2) is a widely studied transcriptional regulator, and most of its downstream genes have antioxidant capacity [26,27]. In our results, there was no significant difference in NFE2L2 regulation of anti-oxidant enzymes between cells with or without RNA2.7 after treatment with Erastin (*z*-score: 2.13 vs. 2.67). The expression of molecules previously identified to regulate ferroptosis-related genes, such as NFE2L2, P53 and NCOA4, were not affected by RNA2.7 (S4B–S4C Fig). We speculated that HCMV RNA2.7 could modify the transcription of genes involved in ferroptosis-related pathways without regulating the activity of NFE2L2.

To analyse ferroptosis related pathways, Gene Set Enrichment Analysis (GSEA) was adopted while pathway enrichment levels were evaluated based on the Erichment score (NES). A |NES value| > 1.0 indicated significant enrichment of genes in the pathway [28]. Fig 3B illustrates that genes associated with iron ions transportation were significantly enriched in 293_RNA2.7_ cells treated with Erastin (NES value: 1.01). Moreover, genes involved in response to iron ions, response to oxidative stress and cystine metabolism were significantly enriched in 293 cells treated with Erastin (NES value: -1.26, -1.34 and -1.16, respectively).

Erastin-induced ferroptosis is characterized by an imbalance between Fe^2+^-mediated peroxide production and GPX4-dependent peroxide elimination (Fig 3C). To investigate the mechanism underlying RNA2.7’s anti-ferroptotic function, we evaluated the key molecules involved in the regulation of iron storage and GPX4 activity in Erastin-treated 293 and 293_RNA2.7_ cells (Fig 3D). No significant difference was observed in the telomeric repeat binding factor 1 (TFR1), recombinant divalent metal transporter 1 (DMT1) and GPX4 proteins between cells with or without RNA2.7. Although the protein levels of FTH1, Ferritin Light Chain (FTL) and SLC7A11 were slightly increased in 293 cells following Erastin treatment, the expression levels of FTH1, FTL and SLC7A11 were increased by 2.88 ± 0.52, 4.59 ± 0.57 and 1.79 ± 0.37 times, respectively in 293_RNA2.7_ cells compared to levels in 293 cells with Erastin. Moreover, the increase in expression level of FTH1 and SLC7A11 in HCMV RNA2.7 exhibited a dose-dependent pattern (Fig 3E).

FTH1 is a component of ferritin that catalyzes the transformation of Fe^2+^ into Fe^3+^ and promotes iron storage [29]. RNA2.7 did not change the total intracellular iron content (Fig 3F). Results showed that RNA2.7 did not influence the transportation of iron ions between extracellular matrix and cytoplasm. To determine if RNA2.7-mediated intracellular Fe^2+^ reduction was due to FTH1 upregulation, 293RNA2.7 cells were transfected with FTH1-targeting siRNAs and treated with Erastin (Fig 3G). FTH1 knockdown abolished the RNA2.7-induced decrease in Fe^2+^ levels following Erastin treatment. In contrast to control cells, Fe^2+^ levels still increased by 1.83±0.42-fold in RNA2.7-expressing cells after Erastin exposure, even with FTH1 knockdown (Fig 3H). These results indicated that HCMV RNA2.7 facilitated intracellular iron storage by increasing FTH1 expression to inhibit ferroptosis.

On the other hand, GPX4 is a GSH-dependent reductase that plays a role in the clearance of peroxides, and SLC7A11 mediates cysteine transportation through system Xc- to promote the synthesis of GSH [15]. To verify whether the increase of SLC7A11 expression by RNA2.7 could alleviate the dysfunction of GPX4 in ferroptosis, two more ferroptosis inducers, Sulfasalazine (SAS) and Buthionine sulfoximine (BSO), were applied. Fig 3C shows that similar to Erastin, SAS induces ferroptosis by suppressing cystine uptake through SLC7A11. Conversely, BSO inhibits GSH synthesis by targeting Glutamate-Cystine Ligase (GCL). The levels of GSH were measured in 293 and 293_RNA2.7_ cells treated with different inducers. Expression of HCMV RNA2.7 abolished the decrease of GSH level induced by Erastin and SAS, which target the transportation of cysteine. However, these effects were not observed in BSO-treated 293_RNA2.7_ cells (Fig 3I). We speculated that, by increasing SLC7A11 level, HCMV RNA2.7 might promote the transportation of cysteine and enhance GPX4 directed clearance of peroxides thereby inhibit ferroptosis.

### Pooled genome-wide CRISPR showed that HCMV RNA2.7 inhibited ferroptosis by targeting ZNF395

**Fig 4:**
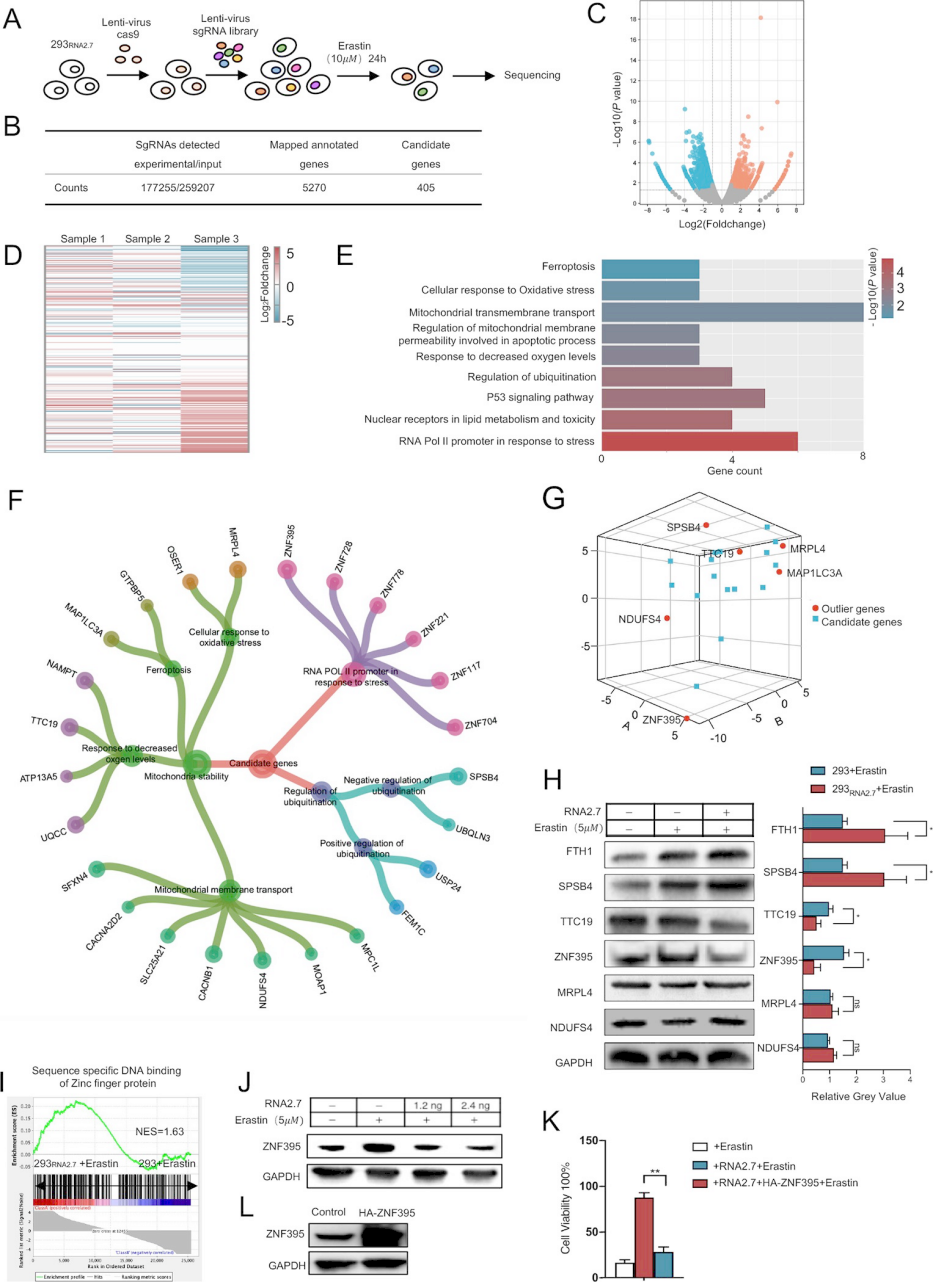
Pooled genome-wide CRISPR showed that HCMV RNA2.7 inhibited ferroptosis by targeting ZNF395. (A) Pooled genome-wide CRISPR screen process was designed to investigate candidate genes involved in ferroptosis regulation by RNA2.7. (B) Counts of sgRNAs obtained from pooled genome-wide CRISPR screen were listed. (C) Candidate genes were significantly enriched with |Foldchange|>2 and *P* value< 0.05 and presented by volcano plots. (D) Heatmap showed Log_2_ (Foldchange) distribution of candidate genes compared with inputs. (E) Candidate genes were enriched by GO analysis. Significantly enriched pathways were presented with histogram indicating gene counts and -Log_10_ (*P* value) of each pathway. (F) Pathways enriched by GO analysis were categorized into 3 aspects: RNA Pol II promoter in response to stress, regulation of ubiquitination and mitochondria stability. Candidate genes enriched in these pathways were showed with different sizes circles indicating RRA scores. (G) RRA scores from three sets of parallel samples were used to label candidate genes within the cube axis and the outlier genes represented higher credibility were marked in red color while other genes were marked in blue. (H) 293 and 293_RNA2.7_ cells were treated with Erastin (5*μ*M) for 24 hours. Regulation effect of RNA2.7 on candidate genes including SPSB4, TTC19, ZNF395, MRPL4 and NDUFS4 were evaluated using Western blots. FTH1 and GAPDH were detected in same samples as positive and load control. Grey value was measured using Image J. Relative Grey value was calculated according to control cells. Error bars show mean±SD for three independent experiments. (I) NES score of sequence specific DNA binding of Zinc finger protein was calculated by GSEA. (J) 293 cells were transfected with 1.2ng and 2.4ng vectors expressing RNA2.7, respectively. At 24 hours post transfection, transfected cells were treated with Erastin (5*μ*M). A dose-dependent regulation of ZNF395 by RNA2.7 was determined using Western blots at 24 hours post treatment. (K) Vectors expressing ZNF395 protein with HA flag was constructed and transfected into 293_RNA2.7_ cells. Overexpression of HA-ZNF395 was validated using Western blots. (L) Cell viabilities of 293 cells, 293_RNA2.7_ cells and 293_RNA2.7_ cells with HA-ZNF395 overexpression were evaluated using viability assay at 24 hours post Erastin (10*μ*M) treatment. Relative viabilities were calculated using normal cells as control. All experiments were conducted independently three times. Error bars represent the mean ± SD from three independent experiments. *: *P* < 0.05; **: *P* < 0.01; ns, no significance.

To identify the target of HCMV RNA2.7 involved in ferroptosis inhibition, pooled genome-wide CRISPR was conducted in 293_RNA2.7_ cells (Fig 4A). Genome-scale CRISPR Knock-Out (GeCKO) v2.0 pooled libraries which contain 259 207 unique sgRNAs targeting 19 050 genes were constructed and transfected into 293_RNA2.7_ cells expressing Cas9 protein [30]. After phenotype stabilization, the 293_RNA2.7_ cells with libraries were treated with Erastin at a final concentration of 10*μ*M for 24 hours. Genomic DNA was extracted from Erastin-resistant cells and subjected to sequencing. An initial screening identified 177,255 sgRNAs mapping to 5,270 genes. Subsequent analysis revealed 405 candidate genes with significant fold changes (|Fold change|> 2) and *P*-values < 0.05 (Fig 4B–4C) [31]. Based on fold change relative to the control group, these candidate genes were categorized into two groups: those whose knockout increased Erastin resistance and those whose knockout increased Erastin sensitivity in 293_RNA2.7_ cells (Fig 4D).

Gene ontology (GO) analysis was performed for candidate genes to determine their biological functions. The results indicated that the candidate genes were mainly enriched in various pathways, such as cellular response to oxidative stress, mitochondrial transmembrane transport and p53 signaling pathway involved in ferroptosis (Fig 4E). Most of these pathways were categorized into 3: RNA Pol II promoter in response to stress, regulation of ubiquitination and mitochondria stability (Fig 4F). The candidate genes in these processes were evaluated based on robust rank aggregation (RRA) scores calculated by Model-based Analysis of Genome-wide CRISPR/Cas9 Knockout (MAGeCK) (Fig 4G) [32]. Outlier genes, including splA/ryanodine receptor domain and SOCS box containing 4 (SPSB4), tetratricopeptide repeat domain 19 (TTC19), zinc finger protein 395 (ZNF395), Mitochondrial ribosomal protein L4 (MRPL4) and NADH Dehydrogenase iron-sulfur protein 4 (NDUFS4) were selected for further validation.

Total proteins were extracted from 293 and 293_RNA2.7_ cells treated with Erastin (10*μ*M) to determine the expression levels of SPSB4, TTC19, ZNF395, MRPL4 and NDUFS4 by Western Blots. Compared to Erastin-treated 293 cells, 293RNA2.7 cells exhibited a 2.73±0.73-fold increase in SPSB4 expression and a 49.64±7.91% and 27.19±11.32% decrease in TTC19 and ZNF395 expression levels, respectively, following Erastin treatment (Fig 4H). There was no significant difference in MRPL4 and NDUFS4 expression between 293 and 293_RNA2.7_ cells. Comparison of the DNA binding activities of ZNFs between 293 and 293_RNA2.7_ cells post-Erastin treatment using GSEA identified a positive NES value of 1.63, indicating that the ZNF activity was altered in cells expressing RNA2.7 (Fig 4I).

Being a member of the C2H2-type zinc finger protein family, ZNF395 is a recently identified nuclear-cytoplasmic shuttling transcription factor that contributes to hypoxia associated inflammation, inhibits immune response and facilitates tumor development [33–37]. Among the 405 identified candidate genes, 5 genes were annotated to proteins that interact with ZNF395 (S5 Fig). Overexpression of RNA2.7 resulted in a dose-dependent reduction of ZNF395 protein levels (Fig 4J). To determine whether downregulation of ZNF395 contributes to RNA2.7-mediated ferroptosis inhibition, ZNF395 was overexpressed in 293_RNA2.7_ cells (Fig 4L). The viabilities of different cells were evaluated after Erastin treatment. The results showed that overexpression of ZNF removed the inhibition of ferroptosis by RNA2.7 (Fig 4K). This indicated that ZNF395 was a target of HCMV RNA2.7 mediating its inhibitory effects on ferroptosis.

### ZNF395 promotes ferroptosis by downregulating FTH1 and SLC7A11 expression

Although ZNF395 was identified as a downstream effector of RNA2.7 in ferroptosis inhibition, its specific role in regulating ferroptosis remains largely unexplored. To investigate ZNF395’s function, we generated ZNF395 overexpression (293_ZNF395oe) and knockdown (293_ZNF395kd) cell lines. These cell lines, along with Erastin-treated 293 and 293_RNA2.7_ cells (as ferroptosis-sensitive and -resistant controls, respectively), were subjected to RNA sequencing analysis.

**Fig 5:**
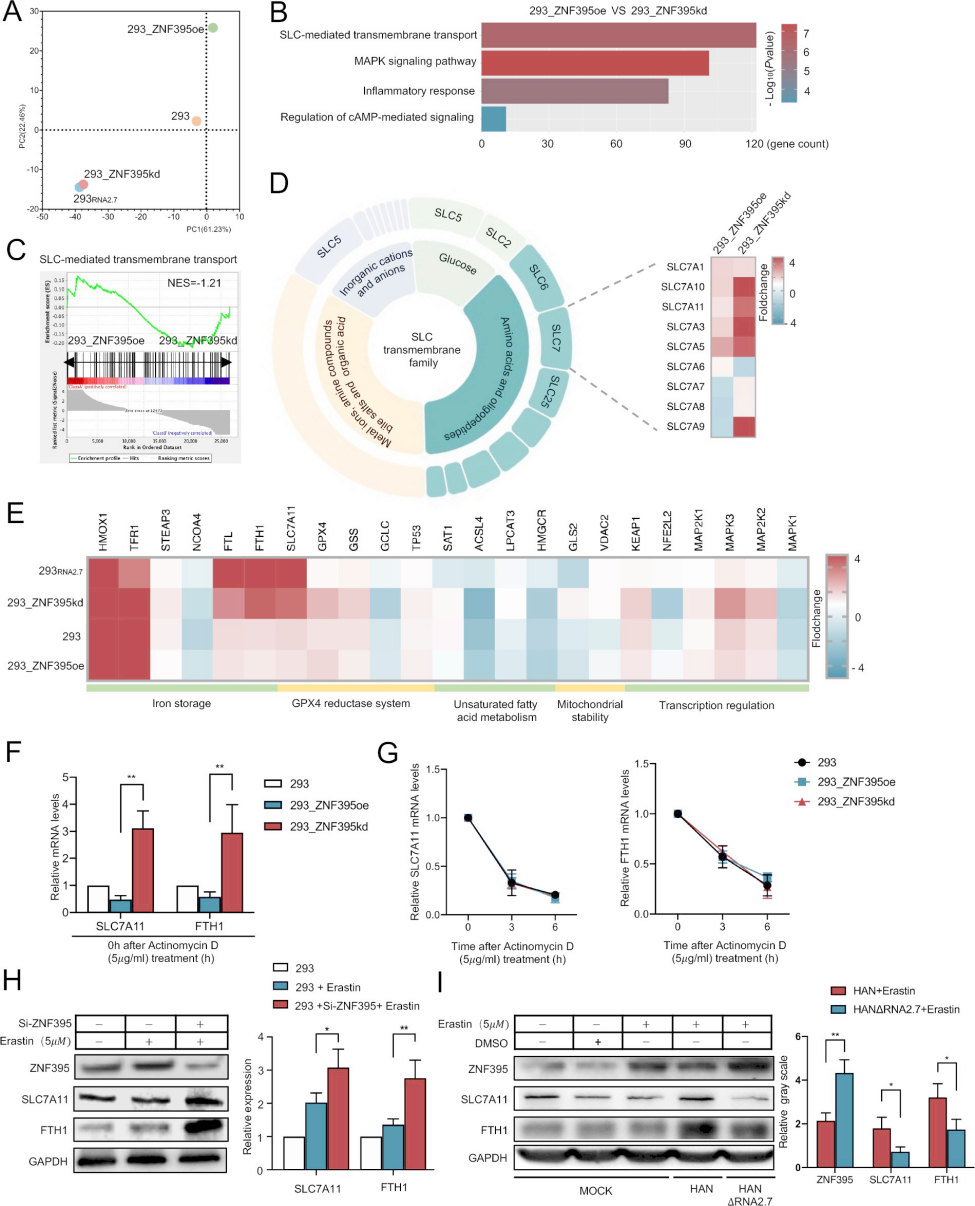
ZNF395 promotes ferroptosis by downregulating FTH1 and SLC7A11 expression. (A) PCA analysis revealed the transcriptomic differences among 293, 293_RNA2.7_, 293_ZNF395oe, and 293_ZNF395kd cells post treatment with Erastin (5*μ*M). 293_ZNF395oe refers to 293 cells with ZNF395 overexpression; 293_ZNF395kd refers to 293 cells with ZNF395 knockdown. (B) Differentially expressed genes between 293_ZNF395oe and 293_ZNF395kd were enriched in several pathways by Go analysis. Significantly enriched pathways were presented with histogram indicating gene counts and -Log_10_ (*P* value) of each pathway. (C) The NES score of pathway SLC-mediated transmembrane transport was calculated using GSEA software. (D) Circular map presented the classification of SLC-transmembrane family. Expression changes of proteins belonged to SLC7 family in 293_ZNF395oe and 293_ZNF395kd cells post Erastin treatment was presented with heatmap. (E) Expression changes of ferroptosis related genes among 293, 293_RNA2.7_, 293_ZNF395oe and 293_ZNF395kd cells post Erastin (5*μ*M) treatment were presented with heatmap. (F) The expression levels of FTH1 and SLC7A11 in 293, 293_ZNF395oe and 293_ZNF395kd cells before Actinomycin D treatment were assessed by qRT-PCR analysis. Each quantative PCR reaction was performed in triplicates, and the results for the target gene mRNA were normalized to GAPDH using the 2^ΔΔCT^ method. The relative mRNA levels were calculated according to 293 cells. The results were presented as means±SD. (G) The mRNA levels of FTH1 and SLC7A11 in 293, 293_ZNF395oe and 293_ZNF395kd cells were assessed at 3 and 6 hours post Actinomycin D treatment (5*μ*g/mL) by qRT-PCR. Each quantative PCR reaction was performed in triplicates, and the results for the target gene mRNA were normalized to GAPDH using the 2^ΔΔCT^ method. The relative mRNA levels were calculated according to cells treated with Actinomycin D 0h. The results are presented as means±SD. (H) 293 cells were transfected with siRNAs targeting ZNF395 (Si-ZNF395) at a final concentration of 100*n*M. Cells were treated with Erastin (5*μ*M) 24 hours post transfection and the expression of SLC7A11 and FTH1 was determined at 24 hours post Erastin treatment by Western blots. GAPDH were detected as load control. Grey value was measured using Image J. Relative Grey value was calculated according to control cells. Error bars show mean±SD for three independent experiments. (I) HELF cells were infected with HAN or HANΔRNA2.7 (MOI = 1) for 24 hours. Erastin (5*μ*M) was added into the supernatants respectively and DMSO was treated as vehicle control. The expression of FTH1, ZNF395 and SLC7A11 was determined at 24 hours post treatment. GAPDH was detected as loading control. Grey value was measured using Image J. Relative Grey value was calculated according to control cells. Error bars represent the mean ± SD from three independent experiments. *P <0 .05; **P <0 .01.

Principal Component Analysis (PCA) was performed to compare the transcriptomic profiles of the four groups for various clusters of 293_RNA2.7_ and 293_ZNF293kd cells. There was significant separation between 293_RNA2.7_ and 293_ZNF395oe cells. Notably, the results revealed that the 293_RNA2.7_ and 293_ZNF395kd cells exhibited similar transcriptomic profiles after Erastin treatment, while the transcriptome profiles of 293_ZNF395oe were significantly different from those of 293_RNA2.7_ and 293_ZNF395kd (Fig 5A).

Differently expressed genes between Erastin-treated 293_ZNF395oe and 293_ZNF395kd were significantly enriched in pathways related to ferroptosis regulation, particularly solute carrier (SLC)-mediated transmembrane transport (Fig 5B). The significantly altered genes were enriched in SLC-mediated transmembrane transport in 293_ZNF395kd cells with an NES value of -1.21 (Fig 5C). The SLC transporter families are nutrient transporters, which function as metabolic gates in various cells [38]. Knock down of ZNF395 significantly increased the transcription of genes belonging to the SLC7 family, which regulate the transportation of amino acids and oligopeptides, including SLC7A11 (Fig 5D).

The transcription of genes involved in ferroptosis-regulatory processes which contained iron storage and GPX4 reductase system was compared among different cells (Fig 5E). The results showed that Erastin activated the transcription of Heme oxygenase 1 (HMOX1) and TFR1 in all groups. Overexpression of RNA2.7 upregulated the mRNA levels of FTL, FTH1, and SLC7A11 significantly. Moreover, ZNF395 knock down enhanced the expression of FTL, FTH1, SLC7A11, Mitogen-activated protein kinase 3 (MAPK3) and Mitogen-activated protein kinase 2 (MAP2K2). Knock down of ZNF395 led to a significant increase of relative FTH1 and SLC7A11 mRNA levels, which was further confirmed to have no impact on mRNA stability by actinomycin D assay (Fig 5F–5G). This implied that ZNF395 might inhibit the transcription of FTH1 and SLC7A11.

Western blot analysis confirmed that ZNF395 represses FTH1 and SLC7A11 expression in 293 cells (Fig 5H). Knock down of ZNF395 using siRNA resulted in a 1.51±0.29-fold increase in FTH1 expression and a 2.03±0.17-fold increase in SLC7A11 expression. The correlations among RNA2.7, ZNF395, FTH1 and SLC7A11 were further investigated in HELF cells infected with HAN or HANΔRNA2.7 (Fig 5I). Compared with the HAN-infected cells, the absence of RNA2.7 led to a 117.29 ± 11.33% increase in ZNF395 expression, accompanied by a 25.69 ± 6.89% and 52.78 ± 13.23% decrease in FTH1 and SLC7A11 protein levels, respectively. These findings suggested that ZNF395 might promote ferroptosis by downregulating FTH1 and SLC7A11 expression.

### A 53nt-in-lenghth sequence in RNA2.7 promotes proteasome-mediated degradation of ZNF395

**Fig 6:**
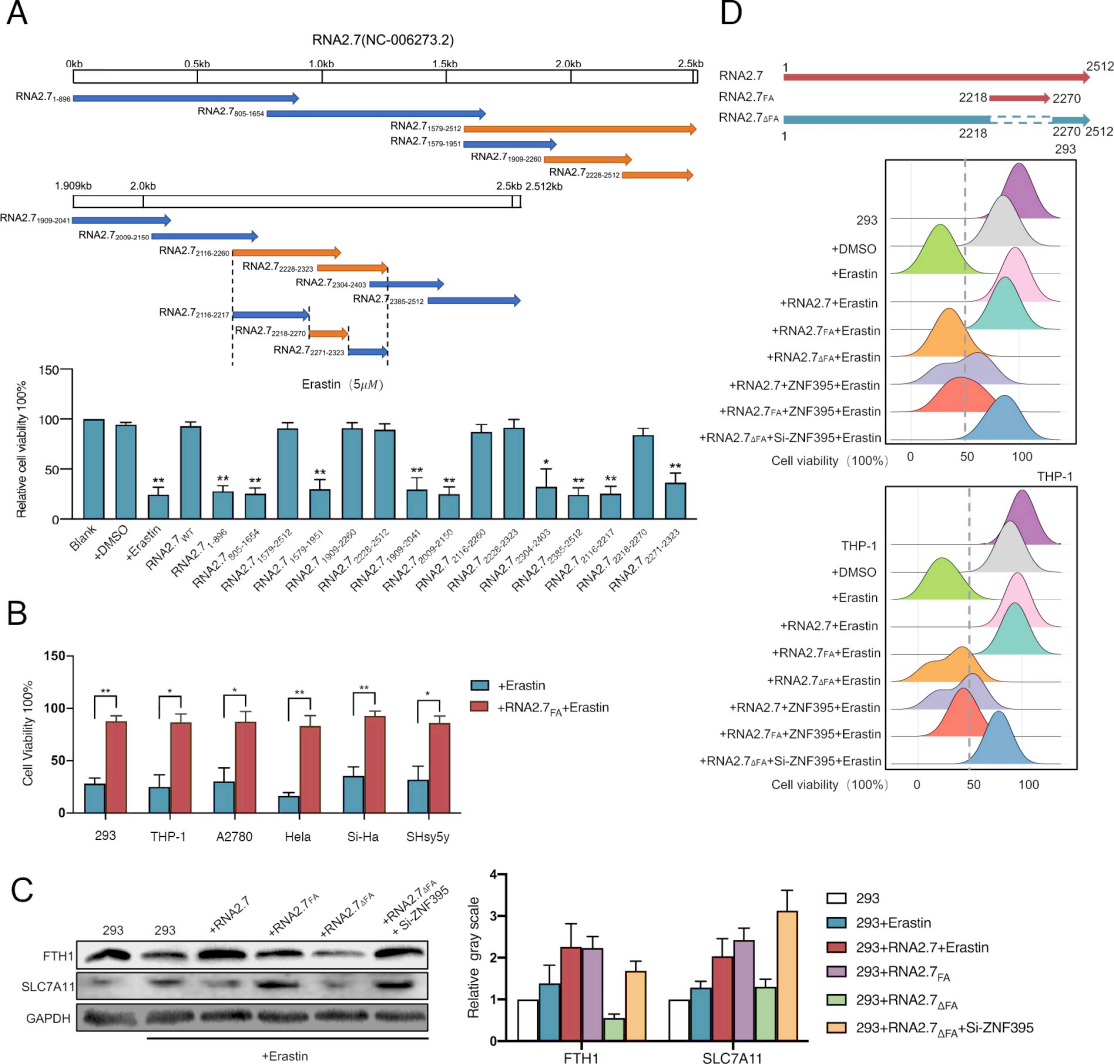
A 53nt-in-lenghth sequence in RNA2.7 functionally inhibits ferroptosis. (A) Selected HCMV RNA2.7 sequences were expressed in 293 cells. Cell viabilities were evaluated using MTS Assay at 24h post Erastin (5*μ*M) treatment. Relative viabilities were calculated using normal cells as control. All experiments were conducted independently three times. Error bars represent the mean ± SD from three independent experiments. A 53nt-in-length sequence in RNA2.7 (located from 2218-2270nt) was found to inhibit ferroptosis and was named as RNA2.7_FA_. (B) 293, THP-1, A2780, Hela, Si-Ha and SHsy5y cells were transfected with vectors expressing RNA2.7_FA_ and treated with Erastin (10*μ*M) at 24 hours post transfection. Cell viabilities were assessed using MTS Assay at 24h post treatment. Relative viabilities were calculated according to control cells. All of the experiments were repeated for three times individually and the Error bars showed mean±SD. (C) The vector expressing RNA2.7_FA_ and RNA2.7 lack of RNA2.7_FA_ motif (RNA2.7_ΔFA_) were constructed and transfected into 293 cells. The expression of SLC7A11 and FTH1 was determined post 24 hours Erastin (5*μ*M) treatment using Western blots. GAPDH were detected as load control. Grey value was measured using Image J. Relative Grey value was calculated according to control cells. Error bars showed mean±SD for three independent experiments. (D) The vector expressing RNA2.7 and RNA2.7_FA_ were co-transfected with vector expressing ZNF395 or not in 293/THP-1 cells. Vectors expressing RNA2.7_ΔFA_ were co-transfected with Si-ZNF395 or not in 293/THP-1 cells. Cell viabilities were evaluated using cell viability assay at 24 hours post Erastin (5*μ*M) treatment and relative viabilities were calculated according to control cells. All of the experiments were repeated for three times individually and the Error bars showed mean±SD. *, *P* < 0 .05; **, *P* < 0 .01.

To identify the functional motif of RNA2.7 that inhibits ferroptosis, selected HCMV RNA2.7 sequences were expressed in 293 cells. The impacts of functional sequences on the cell viabilities after Erastin treatment were evaluated (Fig 6A). A 53nt-in-length sequence in RNA2.7 (located from 2218-2270nt) was found to inhibit ferroptosis effectively and was named as RNA2.7_Ferroptosis Antagonist_ (RNA2.7_FA_). By alignment comparison, it was found that RNA2.7_FA_ sequence was conserved among HAN, TB40/E and Toledo strains (S10A Fig). Cells expressing RNA2.7_FA_ maintained viability above 84.08 ± 6.75% after Erastin treatment. The role of RNA2.7_FA_ in inhibiting ferroptosis was further confirmed in several cell lines expressing RNA2.7_FA_ sequence. The results showed that the viability of cells expressing RNA2.7_FA_ was higher than that of control groups with a value of 71.92 ± 10.02% after Erastin treatment (Fig 6B).

The vector expressing RNA2.7_FA_ and RNA2.7 lack of RNA2.7_FA_ motif (RNA2.7_ΔFA_) were constructed and transfected into 293 cells. Similar to RNA2.7, expression of RNA2.7_FA_ increased the protein levels of FTH1 and SLC7A11. Transfection only with RNA2.7_ΔFA_ could not influence the expression of FTH1 and SLC7A11. By knocking down the expression of ZNF395, FTH1 and SLC7A11 protein levels were increased significantly (Fig 6C). The regulation effect of RNA2.7_FA_ on ferroptosis through ZNF395 was further verified by comparison of cell viabilities in 293 and THP-1 cells respectively (Fig 6D). It was confirmed from the results that the sequence RNA2.7_ΔFA_ was the functional motif of RNA2.7 that increase the expression of anti-ferroptosis genes, FTH1 and SLC7A11, by repressing ZNF395.

**Fig 7:**
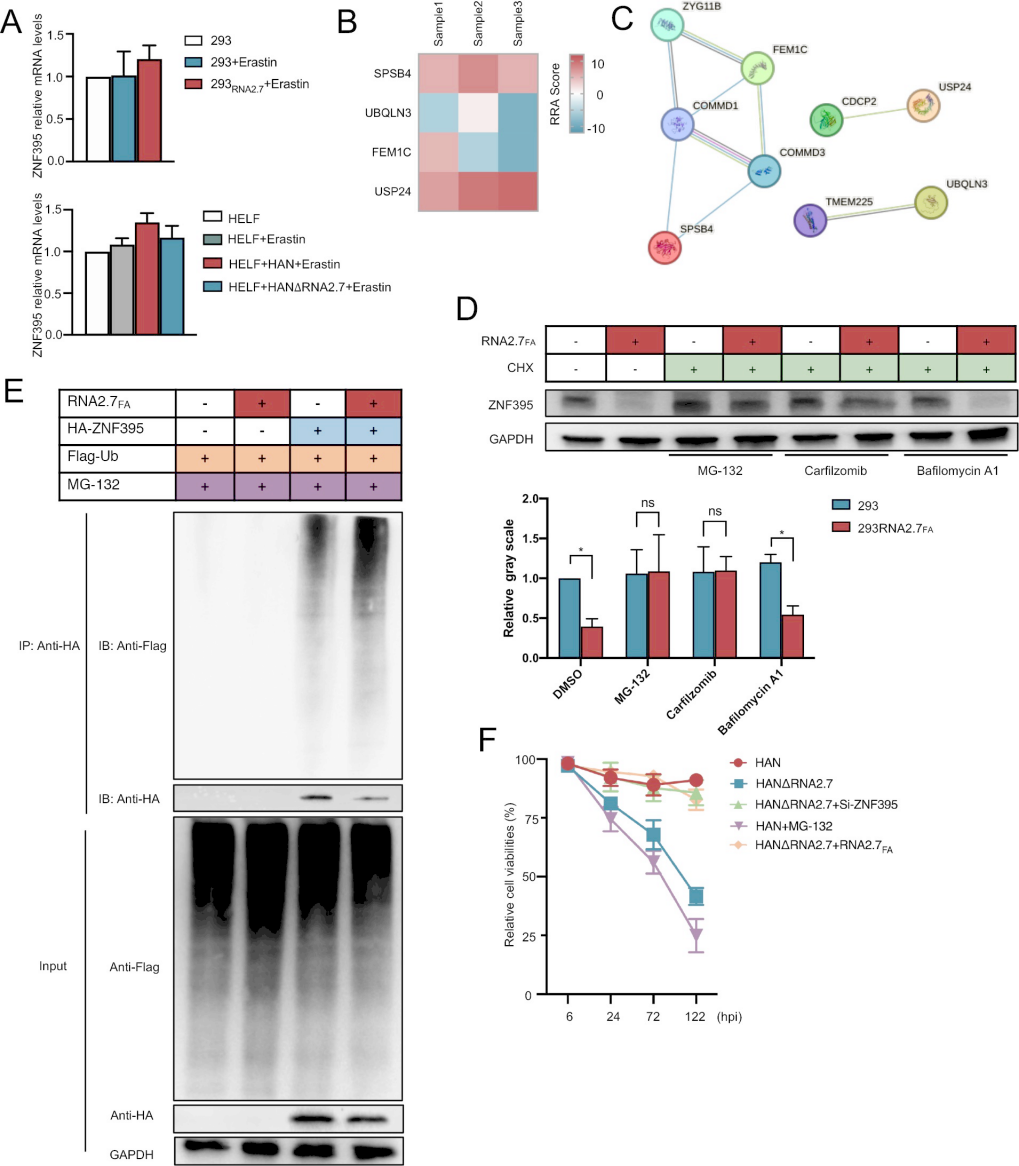
RNA2.7_FA_ sequence promotes proteasome-mediated degradation of ZNF395. (A) ZNF395 mRNA levels were measured in 293 and 293_RNA2.7_ cells treated with Erastin (5*μ*M). So did HELF cells infected with HAN or HANΔRNA2.7 (MOI = 1) treated with Erastin (5*μ*M). Each quantitative PCR reaction was performed in triplicates, and the results for the target gene mRNA were normalized to GAPDH using the 2^ΔΔCT^ method. Results are presented as means±SD. (B) The RRA scores of CRISPR screened candidate genes involved in regulation of ubiquitination, including SPSB4, UBQLN3, FEM1C, and USP24, were presented in a heatmap. (C) Interactions among SPSB4, UBQLN3, FEM1C, USP24 and related proteins were analyzed by STRING. (D) 293 and 293_RNA2.7_ cells were treated with cycloheximide (CHX) at a concentration of 4*μ*M to halt protein synthesis. Following CHX treatment, cells were exposed to MG-132 (100*n*M), Carfilzomib (10*n*M), and Bafilomycin A1 (2.5*n*M) for 8 hours, respectively. ZNF395 protein levels were assessed by Western blots. GAPDH served as a loading control. Grey values were measured by Image J. Error bars show mean±SD for three independent experiments. (E) 293 and 293_RNA2.7_ cells were co-transfected with Ub-Flag and HA-ZNF395 followed by MG132 (10*μ*M) treatment. At 24 hours post transfection, HA-ZNF395 in lysates was immunoprecipitated using anti-HA magnetic beads. Ub-Flag binding to HA-ZNF395 was measured using Western blots with anti-Flag antibody. (F) HELF cells were infected with HAN and HANΔRNA2.7 (MOI = 5). Three additional groups were introduced, including HAN-infected cells with MG-132 treatment (10*μ*M), HANΔRNA2.7-infected cells with RNA2.7_FA_ overexpression, and HANΔRNA2.7-infected cells with ZNF395 knockdown. Cell viabilities were evaluated at 6, 72, 96 and 120 hpi, respectively. Relative viabilities were calculated according to control cells. All of the experiments were repeated for three times individually and the Error bars showed mean±SD. *, *P*<0.05; ns, no significance.

Further analysis found that transfection or infection of RNA2.7 did not alter ZNF395 mRNA levels (Fig 7A). Screening of data from pooled genome-wide CRISPR revealed that genes involved in the regulation of ubiquitylation (such as SPSB4 and USP24) were involved in the inhibitory mechanism of RNA2.7 on ferroptosis (Fig 7B–7C). Studies have reported that ZNF395 is usually degraded through proteasome post ubiquitylation [39]. To address whether RNA2.7 represses ZNF395 by promoting proteasome-mediated degradation, two proteasome inhibitors (MG-132 and Carfilzomib) were added. Lysosome inhibitor Bafilomycin A1 served as the control. After treatments with different inhibitors, it was found that MG-132 and Carfilzomib effectively eliminated the decrease of in ZNF395 protein induced by RNA2.7_FA_, while Bafilomycin A1 did not (Fig 7D). To further confirm the degradation of ZNF395 by RNA2.7_FA_ via proteasomes, vectors expressing HA-ZNF395 were co-transfected with Ub-Flag vectors into 293 and 293_RNA2.7_ cells, respectively. Ubiquitin binding with HA-ZNF395 was immunoprecipitated. Overexpression of RNA2.7_FA_ resulted in enhanced ubiquitin binding to ZNF395 proteins indicating increased activity of proteasome-mediated degradation of ZNF395 by HCMV RNA2.7 (Fig 7E). To address whether RNA2.7 enhanced Ub of ZNF395 through a direct interaction with ZNF395, RNA immunoprecipitation (RIP) was performed to capture RNAs binding to ZNF395. No enrichment of RNA2.7 or RNA2.7_FA_ was found in RNA samples captured by ZNF395 antibody. Thus, no direct interaction was identified between RNA2.7 and ZNF395 (S6 Fig).

**Fig 8:**
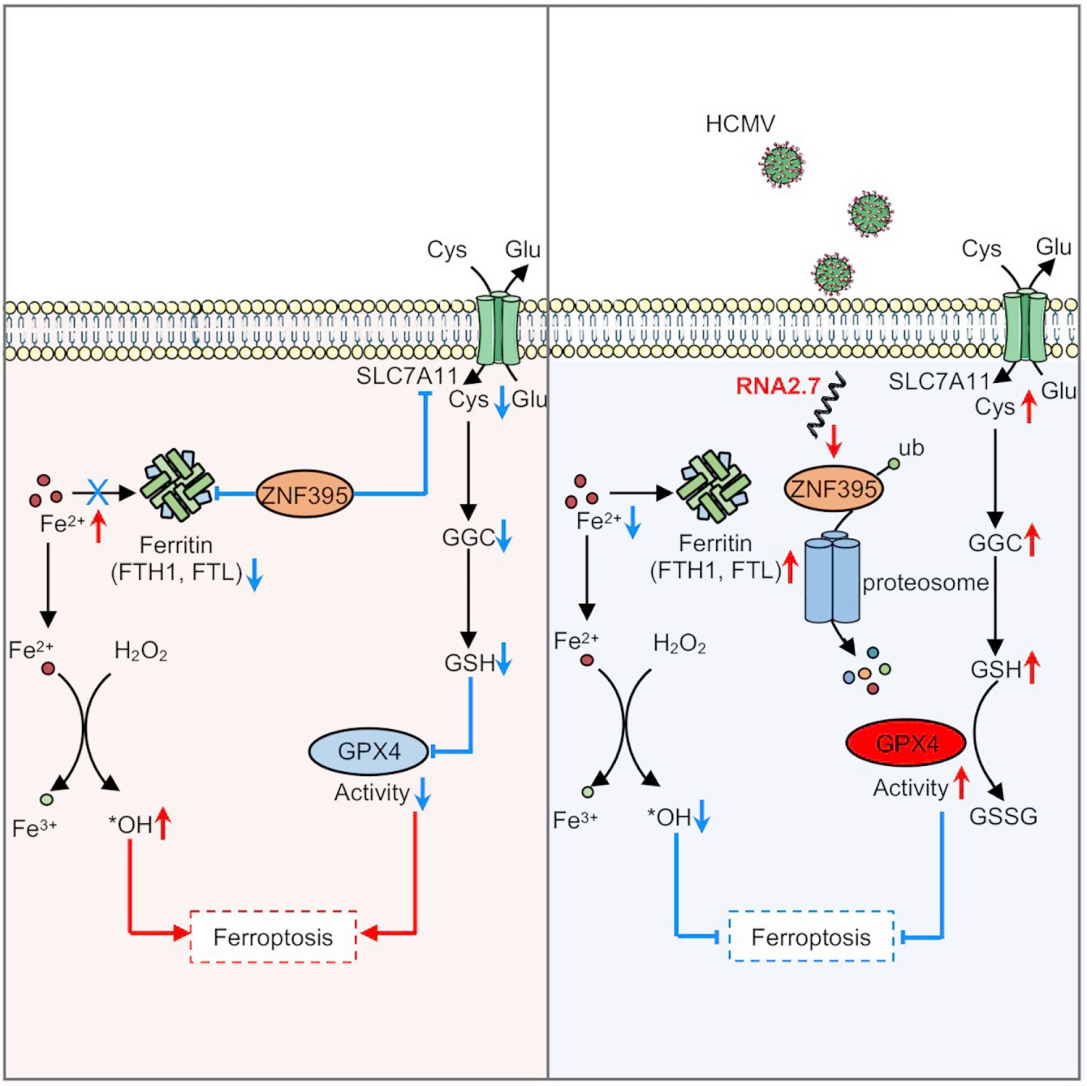
Schematic diagram presents the abstract of mechanism by which RNA2.7 inhibits ferroptosis. HCMV RNA2.7 promoted proteasome-mediated degradation of ZNF395 that resulted in upregulation of FTH1 and SLC7A11 to inhibit ferroptosis.

Moreover, the survival of cells infected with HAN and HANΔRNA2.7 following different treatments was re-evaluated (Fig 7F). Results demonstrated that RNA2.7_FA_ overexpression and ZNF395 protein knock down could rescue the cell death caused by HANΔRNA2.7 infection. Proteasome inhibition using MG-132 significantly enhanced cell death in HAN-infected cells. Through long-term observation, HANΔRNA2.7 lacking of RNA2.7 could not productively replicate under ferroptosis condition. The defect of viral replication could be rescued by ectopical expression of RNA2.7 and RNA2.7_FA_ (S7 Fig). Moreover, ectopically expressed RNA2.7 and RNA2.7_FA_ could also eliminate the accumulation of ROS, MDA and Fe^2+^ in HANΔRNA2.7-infected cells (S8 Fig). Our findings suggest that ZNF395 negatively regulates FTH1 and SLC7A11 expression. Futher more HCMV RNA2.7 promoted proteasome-mediated degradation of ZNF395 that resulted in upregulation of FTH1 and SLC7A11 to inhibit ferroptosis, therefore maintain survival in host cells and complete replication of virus (Fig 8).

## Discussion

Viral infections can trigger or inhibit cell death depending on the viral species [9,40]. Several viruses have been shown to avoid cell death via various mechanisms [41]. As known, viral infection-associated cell death can help the virus spread [23]. Some viruses encode proteins that inhibit cell death thereby facilitate their proliferation [42–45]. HCMV is the largest known herpes virus with a longer replication cycle allowing it to survival for long in host cells [46]. Conversely, HCMV can establish a persistent latent infection within the human host [47]. Maintaining host cell survival is crucial for the establishment and persistence of latency [48]. In fact, preserving infected cell viability is essential for both the lytic and latent phases of the cytomegalovirus life cycle.

HCMV RNA2.7, a virus-encoded long noncoding RNA, has been identified to inhibit apoptosis of infected cells, contributing to the maintenance of viral latency and survival [22,49,50]. Although some work found no significant effect of RNA2.7 on viral growth kinetics in normal conditions (MOI = 0.1), deletion of RNA2.7 impaired virus growth in glucose deprivation condition [21]. Similarly, our results verified an additional function of RNA2.7 in cell death inhibition. HCMV RNA2.7 could inhibit ferroptosis caused by viral infection (MOI = 5) or ferroptosis inducers, and benefit viral replication in ferroptosis conditions. It is noticed that most previous researches in RNA2.7 function are performed with HCMV Toledo strain while ours is mainly with HAN strain [51]. In this case, HAN RNA2.7 was compared with Toledo RNA2.7 side by side to exclude strain specific phenotypes. It is confirmed that HAN RNA2.7 also provides a protection in virus growth from glucose deprivation condition, which is consistent with Toledo (S9 Fig). Moreover, RNA2.7_FA_ sequences that principally functioned in ferroptosis inhibition are entirely conserved among Toledo, TB40/E and HAN (S10A Fig). Another point to be considered is that RL4, which was predicted to encode product with anti-apoptotic activity, is located within RNA2.7[52]. By alignment among multiple HCMV clinical strains, including Toledo, TB40/E, Merlin and HAN, it is found that all clinical strains exhibit insertion mutations within RL4 sequence. These mutations result in truncation of RL4 open reading frame, leading to the loss of RL4 encoded protein within RNA2.7 in clinical strains (S10B Fig). Therefore, HCMV strains containing RNA2.7_FA_ in RNA2.7 sequence might have function in inhibiting ferroptosis.

The mechanism of ferroptosis has three main aspects: the imbalance of intracellular redox system, elevated intracellular Fe^2+^, and high levels of unsaturated fatty acid [12,14,26]. However, analysis of the transcriptomic data did not find evidence for the regulation of unsaturated fatty acid production by RNA2.7. Moreover, deletion of RNA2.7 dysregulated the intracellular redox system and iron metabolism during HCMV infection. Several genes including FTH1, FTL, and SLC7A11, were increased by HCMV RNA2.7.

The decreased sensitivity of cells to ferroptosis may be one of the mechanisms of tumorigenesis [53,54]. Numerous studies have confirmed a close correlation between viral infection and tumorigenesis [55–57]. In recent studies, HCMV gene products were detected in multiple human malignancies, but the mechanism remains unclear [58,59]. HBV regulated ferroptosis of infected cells and caused liver damage by downregulating SLC7A11[60]. Our findings demonstrate that HCMV RNA2.7 upregulates SLC7A11 and suppresses ferroptosis in infected cells. These observations raise the possibility that RNA2.7 may contribute to tumorigenesis by protecting infected cells from ferroptotic cell death. Further investigation is required to elucidate this potential role.

Intracellular levels of SLC7A11 and FTH1 are tightly regulated at both transcription and post-translation levels. These proteins can be regulated by several molecules. TP53 and NFE2L2 activate the transcription of FTH1 and SLC7A11, and NCOA4 increases the abundance of FTH1 by protecting it from ferritinophagy [61–63]. In this study, RNA2.7 did not influence TP53, NFE2L2 and NCOA4 expression. Through pooled genome-wide CRISPR, ZNF395 was found to be the target of RNA2.7 in the regulation of ferroptosis. ZNF395 is a recently discovered nuclear-cytoplasmic shuttling transcription factor that consists of 613 amino acids. It has been shown to activate the transcription of several genes such as CXCL10, as well as modulate immune response and tumor development [33–36]. Our findings reveal a novel suppressive role of ZNF395 on FTH1 and SLC7A11 transcription, a previously undescribed function. We hypothesize that ZNF395 may inhibit FTH1 and SLC7A11 transcription through spatial competition or interference. Further studies are required to elucidate the precise mechanism.

Similar to most viruses, HCMV is strongly related to the host proteome. Studies have reported that HCMV exploits the ubiquitin proteasome system to promote the expression of viral proteins and to manipulate the host proteome in favor of viral replication and immune evasion [64]. For instance, HCMV IE1 has been found to induce ubiquitination of the transcriptional regulator Hes1 and Sp100A [65,66], while pUL69 possesses SUMO E3 ligase activity and hence facilitate the sumoylation of TP53[67]. Although we confirmed HCMV RNA2.7 promoted ZNF395 degradation by enhancing ubiquitination of ZNF395, little is how RNA2.7 achieved this. Moreover, we found SPSB4, which is a member of the E3 ubiquitin ligase family, among CRISPR screened-out candidate genes. The expression of SPSB4 was increased in cells expressing RNA2.7. It provided a possible hypothesis that RNA2.7 might enhance the Ub of ZNF395 via increasing SPSB4. More work is needed to do in further research to solve it.

The interplay between viral infection and ferroptosis has not been sufficiently studied. Recent studies on SARS-CoV-2, HCV, and HIV-1 have suggested a strong association between these two entities [43,68,69]. Viruses can promote ferroptosis by disrupting iron metabolism, antioxidant systems, and host cell immunity via various pathways, which favors virus survival, replication and evasion of host immune attack. Therefore, targeting the regulatory mechanism of ferroptosis by HCMV may be a promising anti-HCMV treatment.

## Materials and methods

### Cells and cell culture

Human embryonic lung fibroblasts (HELF), 293, THP-1, Si-Ha, A2780, HeLa and SHsy5y cells were purchased from Shanghai Institute of Cellular Cells, Chinese Academy of Sciences. HELF, SH and 293 cells were grown in Dulbecco’s modification of Eagle’s medium (DMEM) medium (KeyGEN Biotech) supplemented with 10% Fetal bovine serum (FBS, SinoBiological) in 37°C, 5% CO_2_. THP-1, Si-Ha, A2780, HeLa cells were grown in 1640 medium (KeyGEN Biotech) supplemented with 10% Fetal bovine serum (FBS, SinoBiological) in 37°C, 5% CO_2_. All cells were confirmed to be mycoplasma-negative (Lonza MycoAlert).

### Viruses

HCMV clinical strain (GenBank accession number: KJ426589.1) was isolated from the urine of a 5-month-old patient in Shengjing hospital in 2007[70]. The clinical diagnosis included cytomegalovirus hepatitis, congenital biliary malformation and anemia, and congenital heart disease (aortic stenosis, patent ductus arteriosus, atrial septal defect). The urine HCMV DNA was detected by fluorescence real-time quantitative PCR. HAN-bacterial artificial chromosome (BAC) was constructed and the rescued virus strain was named HAN. RNA2.7 deleted HAN strain named HCMV HANΔRNA2.7 has been used in previous study [51]. HCMV strain TB40/E was given as a gift from professor Hua Zhu of Rutger-New Jersey University.

HAN, TB40/E and HANΔRNA2.7 are cultured in HELF cells, when 90% ~ 100% of the infected cells express GFP, continue to maintain the culture for 2–3 days, blow the cells repeatedly to break the cells and release virus particles for ultracentrifugation collection of virus particles. Virus particles were stored at -80°C until use.

### Plasmid construction

HCMV RNA2.7 sequence was amplified from cDNA using Primer STAR Max DNA Polymerase Mix (TaKaRa). PCR products were inserted into pcDNA3.1 (-) vector (ThermoFisher) and pLJM-1 vector (ThermoFisher). HA-ZNF395 was amplified from cDNA using Primer STAR Max DNA Polymerase Mix (TaKaRa). PCR products were inserted into pcDNA3.1 (-) vector (ThermoFisher). A series of RNA2.7 sequences in different lengths were obtained using different primers. PCR products were inserted into pcDNA3.1 (-) vector (ThermoFisher). ShRNA targeting RNA2.7 (shRNA2.7) was designed by thermofisher. ShRNA2.7 single strand DNA sequences were synthesized by Sangon Biotech. After Annealing, the products were inserted into pLKO.1 vector. Recombinants were transformed into DH5α (TaKaRa).

All of the primers used in the vector construction are listed in S3 Table. All of the constructed plasmids have been sequenced to exclude mutation. Constructed plasmids were extracted by plasmid DNA purification kit (Promega) according to the manufacturer’s instructions.

### Cell viability assay

HELF cells were seeded in 96-well plates and infected with HAN or HANΔRNA2.7 (MOI = 5). Infected cells were treated with chemicals (S3 Table) or transfected with Si-ZNF395 at 24hours post infection. Post 6, 24, 72, 120 hours of infection, 5*m*g/ml MTS solution (Promega) was directly added into medium and incubated for 2–4 hours. Absorbance at wavelengths of 490*n*m was measured. Relative cell viabilities were calculated by comparing the absorbance measured and using mock infected cells as control.

293 cells were transfected with vector expressing different motif of RNA2.7 and 293, THP-1, Si-Ha, A2780, HeLa and SHsy5y cells were transfected with vectors expressing RNA2.7 or RNA2.7_FA_. Ferroptosis inducing chemicals were treated at 24h post infection or transfection (S3 Table). To determine the effect of treatment on cell viability, 5*m*g/ml MTS solution (Promega) was directly added into medium and incubated for 2–4 hours. Absorbance at wavelengths of 490*n*m was measured. Relative cell viabilities were calculated by comparing the absorbance values with untreated cells as control.

### RNA2.7_FA_ delete mutation

A pair of primers containing the reverse complementary sequence across RNA2.7_FA_ sequence was designed according to the pcDNA3.1-RNA2.7 vector sequence (S3 Table). pCDNA3.1-RNA2.7 lacking of RNA2.7_FA_ was amplified by reverse PCR using Primer STAR Max DNA Polymerase Mix (TaKaRa). Then the original plasmid template in the PCR product was digested with DpnI (TaKaRa). The digested product was mixed with 2× MutiF seamless Assembly Mix (ABclonal) according to the manufacturer’s recommendations and kept in 50°C for 15min. Plamids encoding RNA2.7_ΔFA_ of recombination were transformed into DH5α (TaKaRa) and verified by sequencing.

### Lenti-virus preparation

Lentiviral particles were generated through transfection of 293T cells with the lentiviral transfer vector pLJM1-RNA2.7 or pLKO.1-sh-RNA2.7 plus two helper plasmids (VSVG, PSPAX2), using Attractene transfection reagent (Qiagen) according to the manufacturer’s recommendations. Viral supernatant was typically harvested at 48 hours post transfection; cell debris was removed with a 0.22*m*m filter. Viral supernatant was concentrated by PEG8000 (Beyotime Biotechnology) overnight and centrifuged at 4000×g for 20 min. Precipitate was resuspended using DMEM.

### Stable cell line construction

293_RNA2.7_ cells preparation: Day 0: Add 2 × 10^5^ 293 cells in fresh media to 6-well plate. Day 1, Transduction: RNA2.7 lentivirus was diluted in 1 ml infection medium (2% FBS in DMEM) and used to transduce 293 cells. After 6 hours incubation, culture medium was replaced with fresh medium. Two days post infection, remove media containing lentiviral particles from wells and replace with fresh media to a volume of 2*m*L to each well containing puromycin (MCE) to selected wells. Examine viability every 2 days. Complete cell death after 3–5 days should be observed in the control well. Replace the media containing puromycin every 3 days until resistant colonies can be identified and expanded. HELF_sh-RNA2.7_ was constructed in the same way. 293_ZNF395kd was constructed in the same way. 293_RNA2.7_ expressing Cas9 protein was constructed based on 293_RNA2.7_ in the same way.

### PI staining

293 and 293_RNA2.7_ cells were treated with DMSO or 10*μ*M Erastin (MCE) for 24h, The PI staining solution (KeyGen Biotech) was diluted in DMEM at 1:1000 and then added to the cells. After 30min post treatment, supernatant was discarded and cells were washed with PBS for 3 times. The images were captured by fluorescence microscope (Nikon). The magnification for all images presented was 100×.

### ROS assay

293, 293_RNA2.7_, and HELF cells were seeded in 96-well plates. HELF cells were infected with HAN/HANΔRNA2.7 (MOI = 1). Cells were treated with 5*μ*M Erastin (MCE) for 24 hours. To determine the effect of treatment on ROS generation, ROS probe (Beyotime Biotechnology) was added into medium at a concentration of 1:1000 and mix well. 100*μ*l ROS probe solution was directly added for 30min-coincubation at 37°C in dark. Cells were washed by DMEM for 3 times and the fluorescence intensity at wavelengths of 480 *n*m and 525*n*m was measured. Relative ROS levels were calculated by comparing the fluorescence intensity with untreated cells as control.

### Iron assay

293, 293_RNA2.7_, and HELF cells were seeded in 6cm plates. HELF cells were infected with HAN or HANΔRNA2.7 (MOI = 1). Cells were harvested after incubation with 5*μ*M Erastin (MCE) for 24 h. The cells were washed with cold PBS and then homogenized in 4 to 10 volumes of iron assay buffer (Abcam) with 10 to 15 passes or sonicated on ice. The samples were centrifuged at 16000×g for 10 min to remove insoluble materials. A total of 5*μ*l of assay buffer was added to each well to measure Fe^2+^ levels, and 5*μ*l of iron reducer was added to each well to measure total iron levels. Then, the samples were incubated for 30 min at 25°C. After incubation, the iron probe was added, and the cells were incubated for another 60 min at 25°C. Absorbance was measured at 593*n*m by a microplate reader. Relative Iron contents were calculated by comparing the fluorescence intensity with untreated cells as control.

### MDA assay

293, 293_RNA2.7_, and HELF cells were seeded in 6-well plates. HELF cells were infected with HAN or HANΔRNA2.7 at an MOI of 1. Cells were treated with 5*μ*M Erastin (MCE) for 24 hours. MDA content was measured to monitor the lipid peroxidation by MDA Assay kit (Beyotime Biotechnology). Cells were harvested after incubation with Erastin for 24 h. The cells were washed with cold PBS and then homogenized in 320*μ*L MPER buffer (Thermo Fisher) containing protease inhibitors on ice for 1h. The samples were centrifuged at 12000 rpm for 10 minutes at 4°C and 300*μ*l supernatant was used for MDA analysis. Absorbance at wavelengths of 532*n*m and 600*n*m were measured. In the meantime, protein concentration of supernatant was determined by the BCA Protein Assay kit (Takara) after further dilution, and used to normalize the MDA content. Relative MDA contents were calculated by comparing the absorbance values with untreated cells as control.

### Mitochondrial membrane potential assay

293, 293_RNA2.7_, and HELF cells were seeded on glass coverlips and cultured 2–3 days. HELF cells were infected with HAN/HANΔRNA2.7 (MOI = 1) or not. Cells were stained after incubation with 5*μ*M Erastin (MCE) for 24 h. Changes in MMP were assessed using a fluorescence microscope using the directions provided with the JC-1 MMP assay kit (Beyotime Biotechnology). After modeling, the culture medium was removed and each well was washed once with 500*μ*l of Assay buffer. Dilute the JC-1 probes by 200 times with Assay buffer and adds an equal volume of DMEM culture medium. Add the staining solution to cells and incubate at 37°C for 20 minutes. Discard the staining solution and the cells were then washed three times with Assay buffer. A Carl Zeiss LSM880 confocal microscope with NIS Elements was used for image acquisition and analysis. All of the experiments were performed in triplicates, and representative images were shown. The magnification for all images presented is 300×. Fluorescence intensities indicating mitochondrial membrane potential (MMP) were measured utilizing ImageJ software. Relative intensities were calculated according to the mock group as control.

### Transmission electron microscopy

293, 293_RNA2.7_, and HELF cells were used. HELF cells were infected with HAN/HANΔRNA2.7 at an MOI of 1. Cells were harvested after incubation with 5*μ*M Erastin (MCE) for 24 h. Cells (1 × 10^6^ /well) were harvested by centrifugation at 1000 rpm for 5 min. The cells were fixed with 2.5% glutaraldehyde at 4°C for 2 h and washed 6 times with 0.1M phosphate buffer. Then, the cells were fixed with 1% osmium acid for 2 h and washed 3 times with 0.1M phosphate buffer. After that, the cells were fixed with 1% osmium tetroxide and dehydrated through an alcohol gradient before being embedded in resin. The samples were detected using transmission electron microscopy after double staining with uranium acetate and lead citrate. Ultrathin sections (about 60*n*m) were cut on a Reichert Ultracut-S microtome, picked up on to copper grids stained with lead citrate and examined in a JEM 1400Flash transmission electron microscope and images were recorded with an sCMOS camera.

Transmission electron microscopic images and confocal microscopic images were analyzed in ImageJ software [71,72]. Relative fluorescence values of cells in the field of vision were measured and the mean fluorescence value were calculated. Mean fluorescence value = Total fluorescence value of the sight/ Area of the sight. Specifically, tiff formatted transmission electron microscopic images were loaded and read by ImageJ. Scale was first set by the "set scale" option from the "Analyze" tab (1 pixel = 1*n*m for all images). Free hand tool was then used to trace and outline each mitochondrias and all cristae structure. The measurement was stored in the ROI manager. After all mitochondria and the cristae structures were outlined, click "measure" in the ROI manager to get the area measurement. At least 30 mitochondria were outlined. Statistical analyses were performed using GraphPad Prism 5.0. Maximum, minimum, median and upper and lower quartiles of mitochondria and the cristae area were presented.

### Transcriptome sequencing

Cell samples including 293, 293_RNA2.7_, 293_ZNF395oe, 293_ZNF395kd, were harvested post Erastin treatment or not and lysed in TRIzol Reagent (Thermo Fisher) for high throughput RNA sequencing. After RNA quantification and qualification was assessed, a total amount of 1*μ*g RNA per sample was used as input material for the RNA sample preparations. Sequencing libraries were generated using NEBNext Ultra RNA Library Prep Kit following manufacturer’s recommendations and index codes were added to attribute sequences to each sample. Prepared libraries were sequenced on an Illumina Novaseq platform and 150bp paired-end reads were generated.

Index of the reference genome was built and paired-end clean reads were aligned to the reference genome using Hisat2 v2.0.5. Differential expression analysis of two groups (three biological replicates per condition) was performed using the DESeq2 R package (1.16.1). The resulting *P*-values were adjusted using the Benjamini and Hochberg’s approach for controlling the false discovery rate. Genes with Fold Change ≥ 2 and an adjusted *P*-value < 0.05 found were assigned as differentially expressed.

### Quantitative PCR array

293 and 293_RNA2.7_ cells were treated with 5*μ*M Erastin (MCE) for 24 h. Total RNAs was extracted from cultured cells using Total RNA kit (Accurate Biology). Total RNA from each sample was reversely transcribed using Qiagen kit. Reverse transcription was performed at 42°C for 30 min, followed by 70°C for 5 min to inactivate the enzyme activity. Samples were stored at −20°C and subjected to Quantitative PCR arrays (Wcgene Biotechnology) following the instructions of the manufacturer to analyze a panel of ferroptosis-related genes (Wcgene Biotechnology).

### Chemical treatment

Eratin (MCE) was added to HAN or HANΔRNA2.7 infected HELF cells and 293/293_RNA2.7_ cells. RSL3 (MCE) and ML162 (MCE) were used to induce ferroptosis in 293 or 293_RNA2.7_. A series of programmed cell death inhibitors, including z-VAD (MCE), Fer-1 (MCE), DFO (MCE), NAC (MCE) and Necostatin (MCE), were used in HANΔRNA2.7 infected HELF cells to determine forms of cell death regulated by RNA2.7. BSO and Sulfasalazine were added to 293/293_RNA2.7_ cells to validate the effect of RNA2.7 on SLC7A11 respectively. MG-132, Carfilzomib and Bafilomycin A1 were used in 293 cells and 293 cells expressing RNA2.7_FA_ to determine the regulation in proteasome-mediated degradation of ZNF395 by RNA2.7_FA_. Targets and working concentrations of chemicals were listed in S4 Table.

### Western blots

Cells were harvested by centrifugation at 12,000rpm for 5 min at 4°C and lysed in MPER buffer containing protease inhibitors. Protein concentrations were determined using a BCA protein assay kit. After boiling for 10 min in loading buffer, the samples (10*μ*g protein per lane) were separated by 12% SDS-PAGE (Beyotime Biology). The proteins were transferred to nitrocellulose membranes with 0.2-*μ*m diameter pores. The membranes were blocked with 5% nonfat dry milk for 2 h at room temperature and then incubated with primary antibodies, including FTH1 (1:1000, Abcam), FTL (1:1000, Protein tech), GPX4 (1:1000, Abcam), GAPDH (1:10000, Protein tech), SLC7A11 (1:1000, Abcam), DMT1(1:1000, Abcam), TFR1(1:1000, Abcam), SPSB4(1:1000, Abcam), TTC19 (1:1000, Abcam), ZNF395(1:1000, Thermo Fisher), MRPL4(1:1000, Abcam), NDUFS4 (1:1000, Abcam), HA-tag (1:1000, Protein tech), Flag-tag (1:10000, Protein tech), NCOA4 (1:1000, Abcam), NFE2L2 (1:1000, Abcam), pP53 ser6 (1:1000, Abcam), Caspase-3 (1:1000, Abcam), Cytochrome C (1:10000, Abcam) antibodies. After incubation overnight, the membranes were washed 3 times in PBST and incubated with HRP-mouse or HRP-Rabbit antibody (1:10000; Cell Signaling Technology) for another 2h at room temperature. The protein bands were visualized and Densitometric analyses of the bands were performed using ImageJ software. All data are representative of at least 3 independent experiments.

### siRNA transfection

Cells were seeded and Si-FTH1, Si-ZNF395 (Ribo life science) RNA was transfected by Hiperfect transfection reagent (Qiagen) at a final concentration of 200*n*M following the instructions of the manufacturer. The culture medium was replaced 24h after transfection. Knockdown efficiency of FTH1 and ZNF395 was detected by Western blots.

### GSH&GSSG assay

The intracellular concentrations of GSH in 293 cells were determined using GSH&GSSG kit (Beyotime Biology). Cells were harvested by centrifugation at 12000 rpm for 5 min at 4°C and lysed in Protein removal buffer M by freezing and thawing in liquid nitrogen and 37°C water bath 3 times. The samples were centrifuged at 10000×g for 10 min to remove insoluble materials. After mixing the samples with the homogenate reagent for approximately 30 min at room temperature, the yellow colour of the resulting reaction could be observed. The absorbance at 412*n*m was measured using a spectrophotometer. The intracellular concentration of GSH was expressed as *μ*M per gram of protein. Relative GSH&GSSG contents were calculated by comparing the absorbance values with untreated cells as control.

### Pooled genome-wide CRISPR screen

Hundred million 293_RNA2.7-Cas9_ cells were transduced with the Genome-scale CRISPR Knock-Out (GeCKO) v2.0 pooled libraries to achieve 80% infection rate and average 1000-fold coverage of the library after selection. After 24 h, the cells were selected with puromycin and an initial pool of 40 million cells were harvested for genomic DNA extraction using the Qiagen Blood and Tissue extraction kit according to manufacturer protocol. On day 6 post-transduction, 200–400 million 293_RNA2.7-Cas9_ and puromycin resistant 293_RNA2.7-Cas9_ cells were treated with Erastin (10*μ*M) for 24 h. Erastin resistant cells were harvested for genomic DNA. PCR of gDNA was performed in 100*μ*l reactions to attach sequencing adaptors and barcode samples. Samples were quality detected by Agilent 2100 and sequenced on a HiSeq2000 (Illumina).

Raw image Data files obtained by high-throughput sequencing (Illumina sequencing platform) are transformed into Raw sequencing Reads by Base Calling analysis, which is called Raw Data or Raw Reads. The results were stored in FASTQ (fq) file format, which contained the sequence information of the sequencing sequence reads and the corresponding sequencing quality information. The raw sequence obtained by sequencing contains low quality reads with connectors. In order to ensure the quality of information analysis, raw reads need to be finely filtered to obtain clean reads, and subsequent analysis is based on clean reads. According to Illumina sequencing characteristics, using data from two-end sequencing, we required an average Q30 ratio of more than 80% and an average Error rate of less than 0.1%.

The differences between groups were compared. The number of sgrnas that matched perfectly, the average abundance of sgrnas, and the missing sgrnas in the sgRNA library were statistically analyzed. Then the number of enriched sgRNA reads of each type in each sample was counted, and the genes targeted by the sgRNA were annotated. The number of reads enrichment of each gene (genes targeted by different sgrnas) in each sample was counted. The number of reads support corresponding to each sgRNA in each sample was standardized, and the "median" standardization method of MAGeCK was used to standardize the calculation by default. Essential genes were ranked according to the RRA score calculated by MAGeCK, where a higher RRA score indicates a higher importance.

### Quantitative PCR

Total RNA was extracted by using RNA extraction Kit (Accurate) with on column DNase treatment. cDNA was generated from 1*μ*g total RNA using QuantiNova Reverse Transcription Kit (QIAGEN) according to the supplier’s recommendations. Quantitative PCR (qPCR) was performed on QuantStudio Q5 instrument (ThermoFisher). Each 20*μ*L qPCR mixture contained 100ng reverse transcription product, 10 *μ*l 2×QuantiNova SYBR Green PCR Master Mix, 2*μ*L QN ROX Reference Dye (QIAGEN), and 0.7*μ*M forward and reverse primers. Primers used in this study were listed in S2 Table. Amplification was performed by denaturation at 95°C for 2 min, followed by 40 two-step cycles of 95°C for 15s and 60°C for 30s. Each reaction was performed in triplicates, and the results for the target gene mRNA were normalized to GAPDH using the 2^ΔΔCT^ method. The results are presented as means and standard deviation (SD).

### Actinomycin D assay

293 cells were transfected by pLJM-1-HA-ZNF395 vector and Si-ZNF395 RNA and incubated with 2 *μ*g/ml Actinomycin D (Sigma-Aldrich, Steinheim, Germany) to block transcription at 48h post transfection. At 0, 3, or 6 h after Actinomycin D treatment. Cellular RNA was processed by qRT-PCR for quantification of FTH1 and SLC7A11 mRNA level.

### Co-immunoprecipitation

293 and 293_RNA2.7_ cells were transfected with HA-ZNF395 and Ub-Flag (Origene). After 48h transfection, cells were treated with MG-132 (10*μ*M) for 8h. The transfected cells were lysed with MPER Lysis Buffer supplemented with protease inhibitor cocktail on ice before sonicating for 1 min then centrifugation at 12000 × g at 4°C for 10 min. 1/10 volume of the supernatant is retained as input, the remaining was incubated with anti-HA Magnetic beads overnight on a rotator at 4°C. The immunocomplexes were then washed with 500*μ*L of bind & wash buffer 5 times and eluted with SDS buffer.

### RNA immunoprecipitation

The RNA immunoprecipitation (RIP) was performed using the RIP kit (Millipore) according to the manufacturer’s instructions. Briefly, 10^7^ cells were lysed with RIP lysis buffer using one freeze-thaw cycle. Cell extracts were coimmunoprecipitated with anti-HA (CST) and IgG (Millipore) antibodies and the retrieved RNA was subjected to qRT-PCR analysis.

### Sequence alignment

RNA2.7_FA_ in different strains were amplified by PCR by using high fidelity DNA polymerase (Accurate Biology) according to the manufacturer’s instructions. The PCR product was verified by agarose gel electrophoresis. Then the PCR product was inserted into pCR2.1 vector by using the TA-clone kit (Thermo) and the recombinants were transformed into DH5α (TaKaRa). Sangon Bitotech performed DNA sequences.

### Virus growth kinetics

HELFs were infected with HAN/HANΔRNA2.7 at MOI of 1. Genome DNA were collected at 6, 72, 96 and 120 hpi by DNA extraction kit (Tiangen). The relative DNA copy numbers of HCMV genome were measured using quantitative PCR following the instructions of the manufacturer (Qiagen) on QuantStudio Q5 instrument (Thermo Fisher). UL83 was used to indicate the DNA copy number of HCMV genome, and the results for the UL83 DNA were normalized to GAPDH using the 2^ΔΔCT^ method. Three independent experimental replicates were set at each time point and the results are presented as means and SD.

### Pathway analyses

Differentially expressed genes and candidate genes selected from CRISPR screen were functionally categorized and analyzed using Ingenuity Pathway Analysis software (QIAGEN Ingenuity System), Gene Set Enrichment Analysis (GSEA, http://www.broadlinstitute.org/gsea) and Metascape, (https://metascape.org/gp/index.html). The analysis results were analyzed and plotted using the ggplot2 package of R language. Protein interactions were analyzed by STRING (https://cn.string-db.org/).

### Statistical analyses

Statistical analyses were performed using Excel and GraphPad Prism 5.0. Data were expressed as mean ± SD. Statistically significant differences were evaluated using unpaired 2-tailed Student’s t test. In all cases, *P*-value of ≤ 0.05 was considered statistically significant.

## Supporting information

S1 Table Candidate genes by Genome-wide CRISPR Screen. (XLSX)

S2 Table Oligonucleotides used for amplification. (XLSX)

S3 Table Oligonucleotides used for vector clone. (XLSX)

S4 Table Information of chemicals used in this study. (XLSX)

S1 Fig HCMV RNA2.7 inhibits ferroptosis. (A) HELF cells were infected with HAN/HANΔRNA2.7 and treated with Erastin (5*μ*M) at 48hpi. Caspase-3 and Cytochrome C were assessed by Western blots. GAPDH served as loading control. (B) HELF cells were infected with HAN/HANΔRNA2.7. At 6hpi, cells were treated with DMSO/ DFO (100*μ*M) or NAC (10*μ*M) respectively. Post 48 hours treatment, Cells were treated with Erastin (10*μ*M) or not. Cell viabilities were evaluated using cell viability assay at 72hpi. Relative viabilities were calculated using uninfected HELF cells as control. All experiments were conducted independently three times. Error bars represent the mean ± SD from three independent experiments. (C) HELF and HELF expressing Sh-RNA2.7 (HELF_sh-RNA2.7_) cells were infected with TB40/E (MOI = 1). At 24hpi, Total RNA was extracted and quantitative reverse transcription-PCR was assessed to determine the levels of RNA2.7. (D) HELF and HELF_sh-RNA2.7_ cells were infected with TB40/E (MOI = 5). At 24hpi, Erastin were added into the supernatants with final concentration of 5*μ*M or 10*μ*M. Cell viabilities were evaluated using cell viability assay at 6, 72, 96 and 120 hpi. Relative viabilities were calculated according to uninfected HELF cells. All experiments were conducted independently three times. Error bars represent the mean ± SD from three independent experiments. (E) HELF cells were infected with HAN/HANΔRNA2.7 (MOI = 1). At 24 hpi, RSL3 was added into the supernatants with a final concentration of 10*μ*M. Contents of reactive oxygen species (ROS), ferrous iron, and malondialdehyde (MDA) were measured using ROS assay, MDA assay and iron assay at 24 hours post treatment, respectively. Relative contents were calculated using mock group as control. All experiments were conducted independently three times. Error bars represent the mean ± SD from three independent experiments. (F) HELF cells were infected with HAN or HANΔRNA2.7 (MOI = 1) and followed by RSL3 treatment (10*μ*M) at 24hpi. Cells were stained with JC-1 probes at 24 hours post treatment. Images were obtained and analyzed via fluorescence confocal microscopy. The magnification of taken pictures were 300×. Left: representative images are shown; scale bars = 100*μ*m; Right: fluorescence intensities indicating MMP were measured utilizing ImageJ software. Relative intensities were calculated according to the mock group as control. Error bars denote mean ± SD. **, *P* < 0.01; ***, *P* < 0.001.
(TIF)

S2 Fig HCMV RNA2.7 prevents cell death from Erastin-induced ferroptosis. 293 and 293_RNA2.7_ cells were treated with Erastin (10*μ*M) and stained with PI at 24h post treatment. Images were captured by fluorescence microscope and red fluorescent cells represent dead cells. scale bars: 1*m*m. The magnification of taken pictures were 100×.
(TIF)

S3 Fig HCMV RNA2.7 inhibits ferroptosis induced by Erastin, ML162 and RSL3. 293 and 293_RNA2.7_ cells were treated with ferroptosis inducers respectively, including Erastin (5*μ*M), ML162 (10*μ*M) and RSL3 (10*μ*M). RSL3 and ML162 are covalent binding inhibitors of GPX4. Cell viabilities were evaluated using cell viability assay at 24 hours post treatment. Relative viabilities were calculated according to control cells. All experiments were conducted independently three times. Error bars represent the mean ± SD from three independent experiments, *: *P*<0.05; **: *P*<0.01.
(TIF)

S4 Fig HCMV RNA2.7 affects transcriptions of ferroptosis-related genes without influencing known regulators. (A) 293 and 293_RNA2.7_ cells were treated with Erastin (5*μ*M). Total RNA was extracted at 24h post treatment. PCR Array assay was assessed to determine the transcription of ferroptosis related genes. Error bars show mean±SD for three independent experiments. (B) and (C) 293 and 293_RNA2.7_ cells were treated with Erastin (5*μ*M). At 24 hours post treatment, p-P53 (ser6), NFE2L2 and NCOA4 were assessed by western blots. GAPDH served as loading control.
(TIF)

S5 Fig Annotated proteins interact with ZNF395. Annotated proteins interact with ZNF395 assessed by STRING database.
(TIF)

S6 Fig HCMV RNA2.7 does not interact with ZNF395 directly. 293 cells were co-transfected with HA-ZNF395 and pcDNA3.1-RNA2.7_FA_/pcDNA3.1-RNA2.7_Δ FA_/pcDNA3.1(-) respectively. At 48 hours post transfection, cells were harvested for immuno-precipitation to capture RNA interacted with HA-ZNF395 and capture RNA interacted with IgG as control. Relative mRNA levels were determined by quantitative PCR. The results represented three independent experiments.
(TIF)

S7 Fig Ectopically expressed RNA2.7 and RNA2.7_FA_ rescue HCMV replication in ferroptosis condition. HELF and HELF_RNA2.7_ cells were infected with HAN or HANΔRNA2.7 at an MOI of 0.1 with or without Erastin (5*μ*M). DNA of infected cells was extracted at different time points (0, 1, 3, 6 and 9 dpi) respectively. Expression levels of HCMV UL83 were measured using quantitative PCR. Each quantitative PCR reaction was performed in triplicates, and the results for the target gene mRNA were normalized to GAPDH using the 2^ΔΔCT^ method. The relative mRNA levels were calculated according to infected cells at 0hpi. The results are presented as mean±SD.
(TIF)

S8 Fig Ectopically expressed RNA2.7 and RNA2.7_FA_ inhibit the increase of ferroptotic hall marks induced by Erastin. HELF cells were infected with HAN/HANΔRNA2.7 (MOI = 1). At 24 hpi, HANΔRNA2.7 infected HELF cells were transfected with vectors expressed RNA2.7/RNA2.7_FA_ respectively. At 48hpi, Erastin was added into the supernatants with a final concentration of 5*μ*M. Contents of reactive oxygen species (ROS), ferrous iron, and malondialdehyde (MDA) were measured using ROS assay, MDA assay and iron assay at 24 hours post treatment, respectively. Relative contents were calculated using mock group as control. All experiments were conducted independently three times. Error bars represent the mean ± SD from three independent experiments.
(TIF)

S9 Fig HANΔRNA2.7 presents growth defect in glucose deprivation condition. Growth of HAN, HANΔRNA2.7 in HELF cells under metabolically stressed condition. PFU, plaque-forming units. Cells were infected with HAN/HANΔRRNA2.7 at MOI = 0.1. At 24 hpi, HAN infected and HANΔR RNA2.7 infected HELF cells were incubated with normal media/ rotenone(5*n*M) or glucose-depleted media. The virus titer was performed by TCID50 assay and Error bars show mean±SD for three independent experiments.
(TIF)

S10 Fig DNA sequences of RNA2.7_FA_ and RL4 are aligned among various viral strains. (A)The RNA2.7FA sequence of the Toledo strain was utilized as a reference, against which the sequences of other strains were compared. Dots indicate no differences from the reference sequence, while any mutant bases are shown accordingly. (B) The RL4 sequence of the AD169 strain was utilized as a reference, against which the sequences of other strains were compared. Dots indicate no differences from the reference sequence, while any mutant bases are shown accordingly.
(TIF)
