# Supplementary figures and images for: Human cytomegalovirus RNA2.7 inhibits ferroptosis by upregulating ferritin and GSH via promoting ZNF395 degradation
Source: PLoS Pathog. 2024 Dec 26;20(12):e1012815. doi: 10.1371/journal.ppat.1012815 (PMC11709246; doi:10.1371/journal.ppat.1012815)

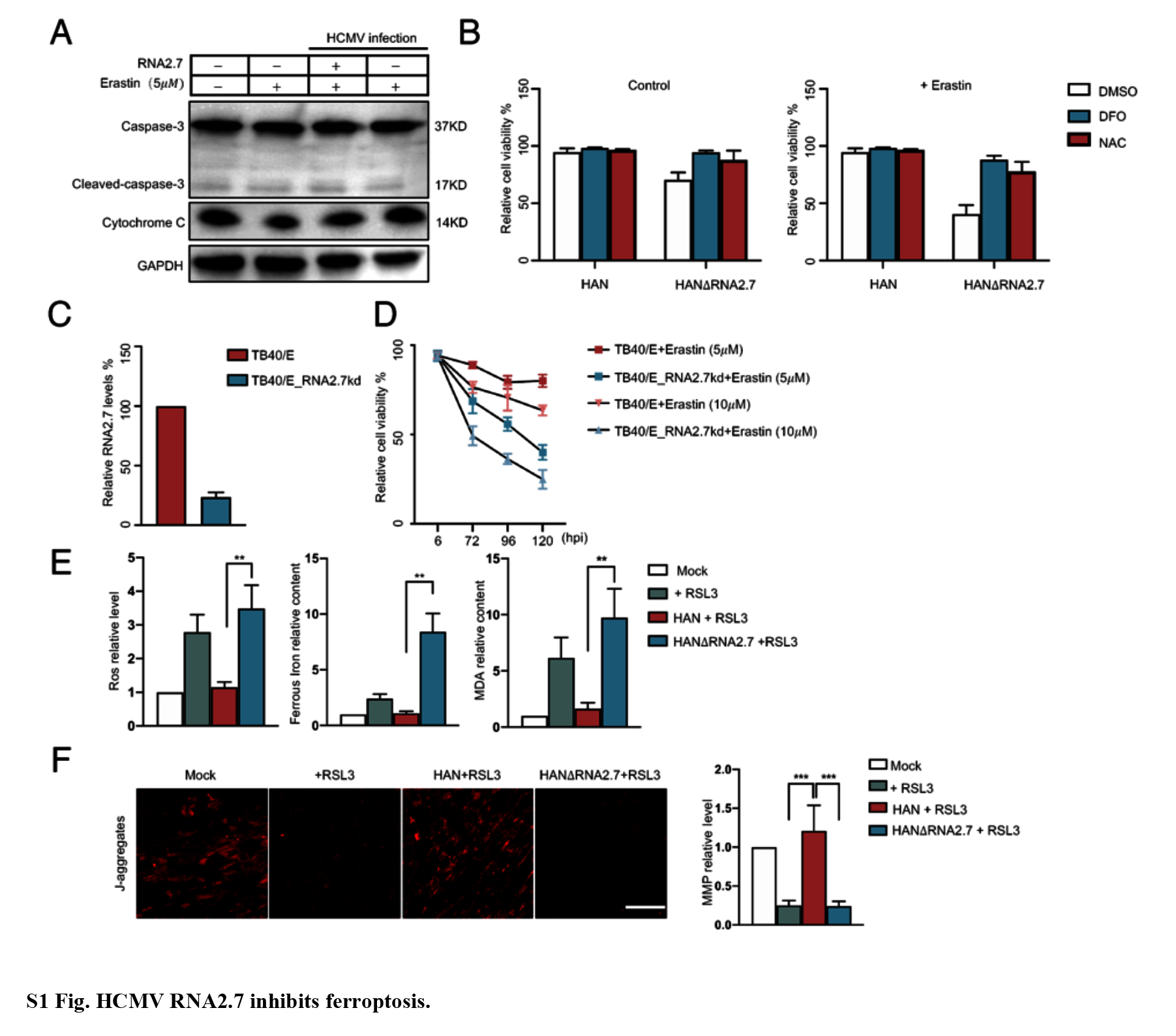

Supplement: S1 Fig — (A) HELF cells were infected with HAN/HANΔRNA2.7 and treated with Erastin (5μM) at 48hpi. Caspase-3 and Cytochrome C were assessed by Western blots. GAPDH served as loading control. (B) HELF cells were infected with HAN/HANΔRNA2.7. At 6hpi, cells were treated with DMSO/ DFO (100μM) or NAC (10μM) respectively. Post 48 hours treatment, Cells were treated with Erastin (10μM) or not. Cell viabilities were evaluated using cell viability assay at 72hpi. Relative viabilities were calculated using uninfected HELF cells as control. All experiments were conducted independently three times. Error bars represent the mean ± SD from three independent experiments. (C) HELF and HELF expressing Sh-RNA2.7 (HELFsh-RNA2.7) cells were infected with TB40/E (MOI = 1). At 24hpi, Total RNA was extracted and quantitative reverse transcription-PCR was assessed to determine the levels of RNA2.7. (D) HELF and HELFsh-RNA2.7 cells were infected with TB40/E (MOI = 5). At 24hpi, Erastin were added into the supernatants with final concentration of 5μM or 10μM. Cell viabilities were evaluated using cell viability assay at 6, 72, 96 and 120 hpi. Relative viabilities were calculated according to uninfected HELF cells. All experiments were conducted independently three times. Error bars represent the mean ± SD from three independent experiments. (E) HELF cells were infected with HAN/HANΔRNA2.7 (MOI = 1). At 24 hpi, RSL3 was added into the supernatants with a final concentration of 10μM. Contents of reactive oxygen species (ROS), ferrous iron, and malondialdehyde (MDA) were measured using ROS assay, MDA assay and iron assay at 24 hours post treatment, respectively. Relative contents were calculated using mock group as control. All experiments were conducted independently three times. Error bars represent the mean ± SD from three independent experiments. (F) HELF cells were infected with HAN or HANΔRNA2.7 (MOI = 1) and followed by RSL3 treatment (10μM) at 24hpi. Cells were stained with JC-1 probes at 24 h [file ppat.1012815.s005.tif]

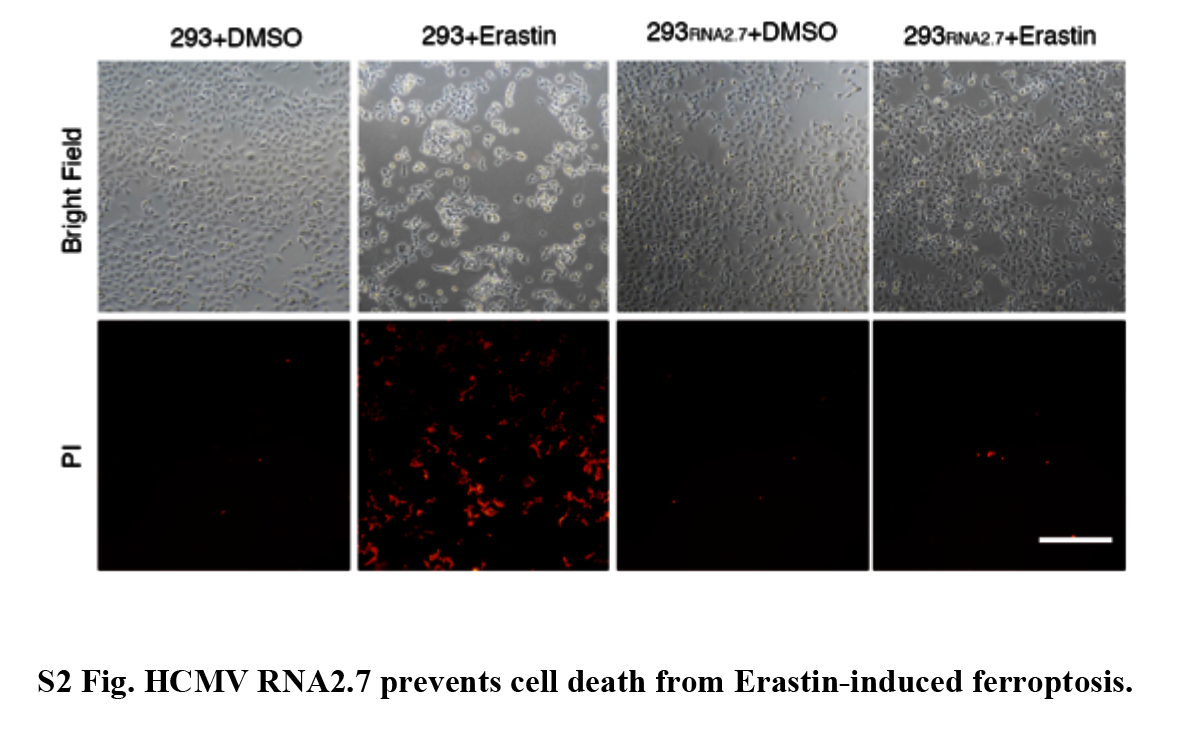

Supplement: S2 Fig — 293 and 293RNA2.7 cells were treated with Erastin (10μM) and stained with PI at 24h post treatment. Images were captured by fluorescence microscope and red fluorescent cells represent dead cells. scale bars: 1mm. The magnification of taken pictures were 100×. (TIF) [file ppat.1012815.s006.tif]

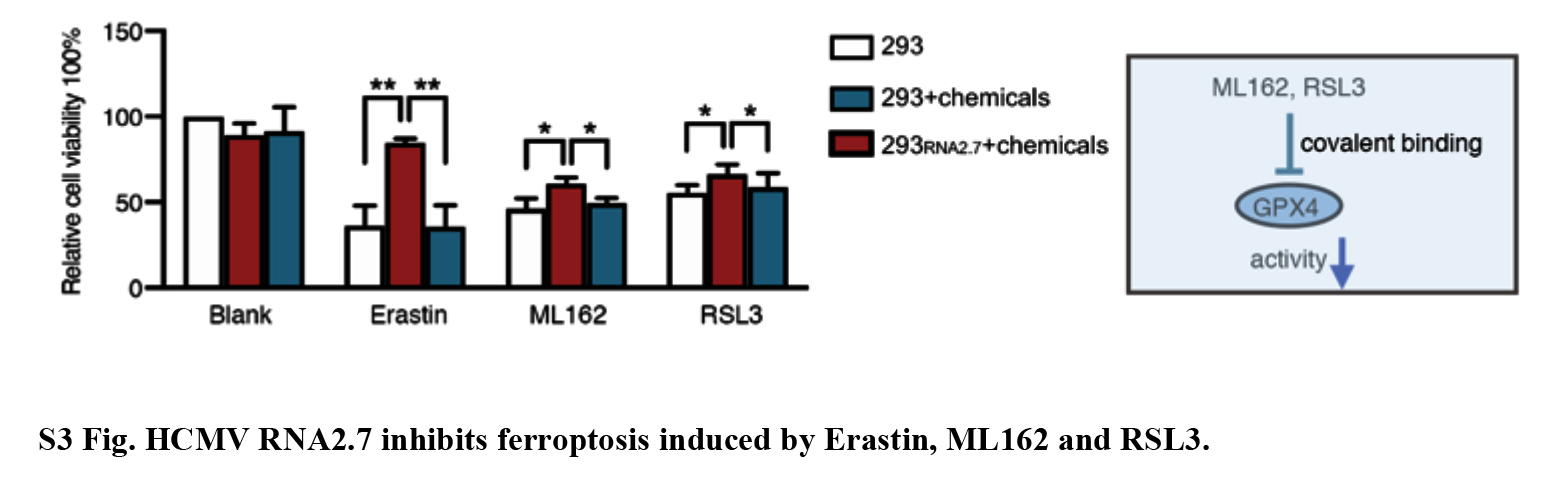

Supplement: S3 Fig — 293 and 293RNA2.7 cells were treated with ferroptosis inducers respectively, including Erastin (5μM), ML162 (10μM) and RSL3 (10μM). RSL3 and ML162 are covalent binding inhibitors of GPX4. Cell viabilities were evaluated using cell viability assay at 24 hours post treatment. Relative viabilities were calculated according to control cells. All experiments were conducted independently three times. Error bars represent the mean ± SD from three independent experiments, *: P<0.05; **: P<0.01. (TIF) [file ppat.1012815.s007.tif]

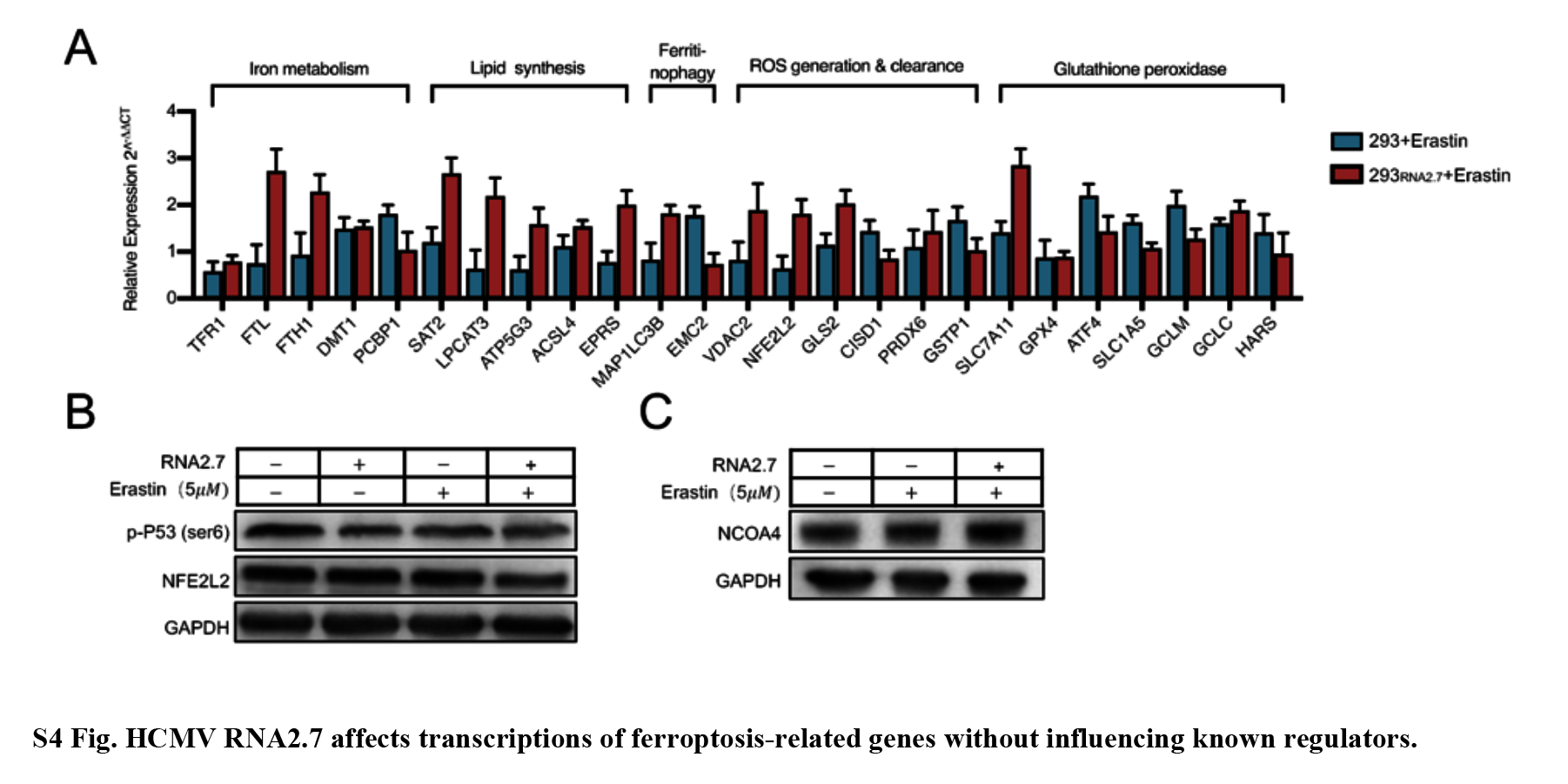

Supplement: S4 Fig — (A) 293 and 293RNA2.7 cells were treated with Erastin (5μM). Total RNA was extracted at 24h post treatment. PCR Array assay was assessed to determine the transcription of ferroptosis related genes. Error bars show mean±SD for three independent experiments. (B) and (C) 293 and 293RNA2.7 cells were treated with Erastin (5μM). At 24 hours post treatment, p-P53 (ser6), NFE2L2 and NCOA4 were assessed by western blots. GAPDH served as loading control. (TIF) [file ppat.1012815.s008.tif]

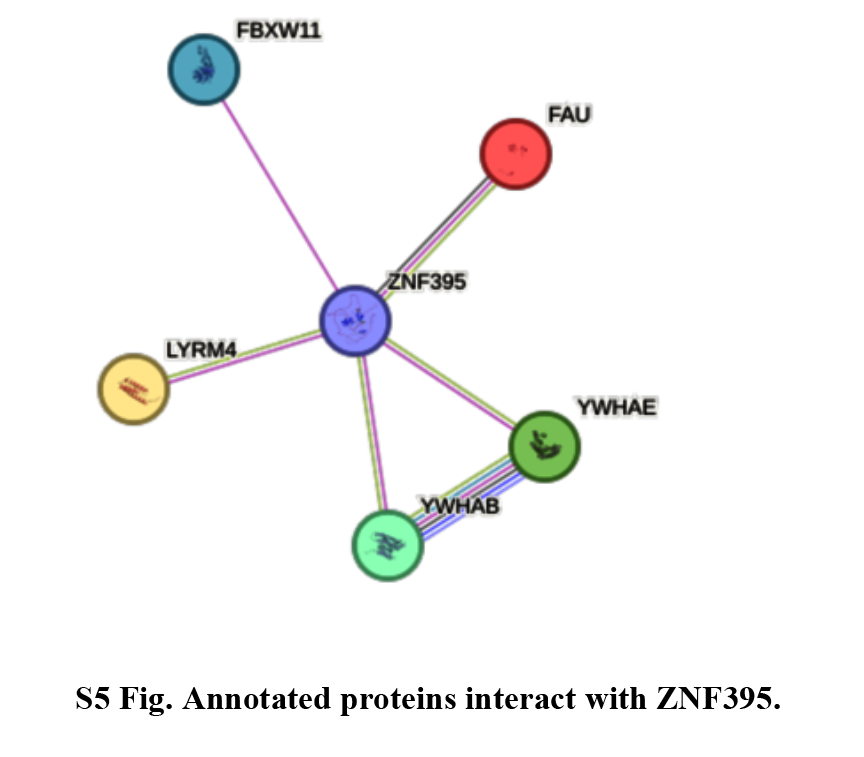

Supplement: S5 Fig — Annotated proteins interact with ZNF395 assessed by STRING database. (TIF) [file ppat.1012815.s009.tif]

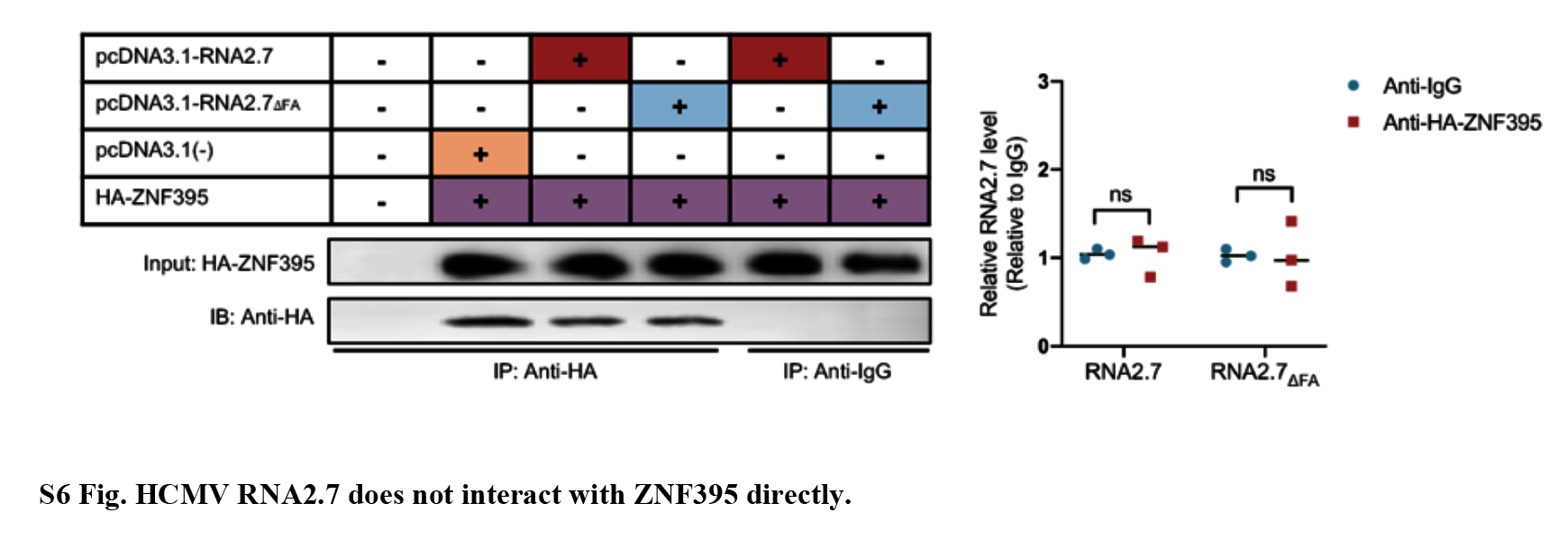

Supplement: S6 Fig — 293 cells were co-transfected with HA-ZNF395 and pcDNA3.1-RNA2.7FA/pcDNA3.1-RNA2.7Δ FA/pcDNA3.1(-) respectively. At 48 hours post transfection, cells were harvested for immuno-precipitation to capture RNA interacted with HA-ZNF395 and capture RNA interacted with IgG as control. Relative mRNA levels were determined by quantitative PCR. The results represented three independent experiments. (TIF) [file ppat.1012815.s010.tif]

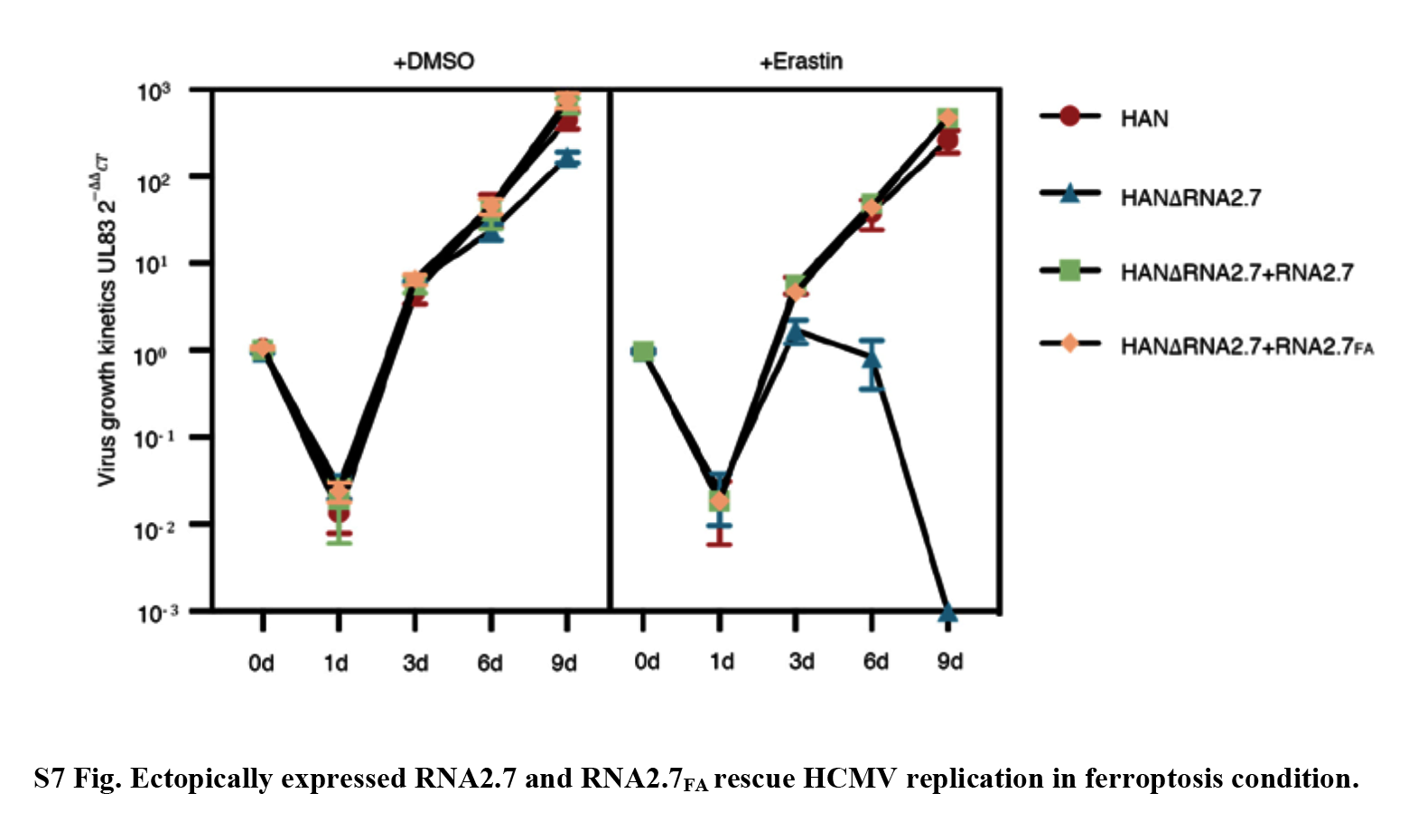

Supplement: S7 Fig — HELF and HELFRNA2.7 cells were infected with HAN or HANΔRNA2.7 at an MOI of 0.1 with or without Erastin (5μM). DNA of infected cells was extracted at different time points (0, 1, 3, 6 and 9 dpi) respectively. Expression levels of HCMV UL83 were measured using quantitative PCR. Each quantitative PCR reaction was performed in triplicates, and the results for the target gene mRNA were normalized to GAPDH using the 2ΔΔCT method. The relative mRNA levels were calculated according to infected cells at 0hpi. The results are presented as mean±SD. (TIF) [file ppat.1012815.s011.tif]

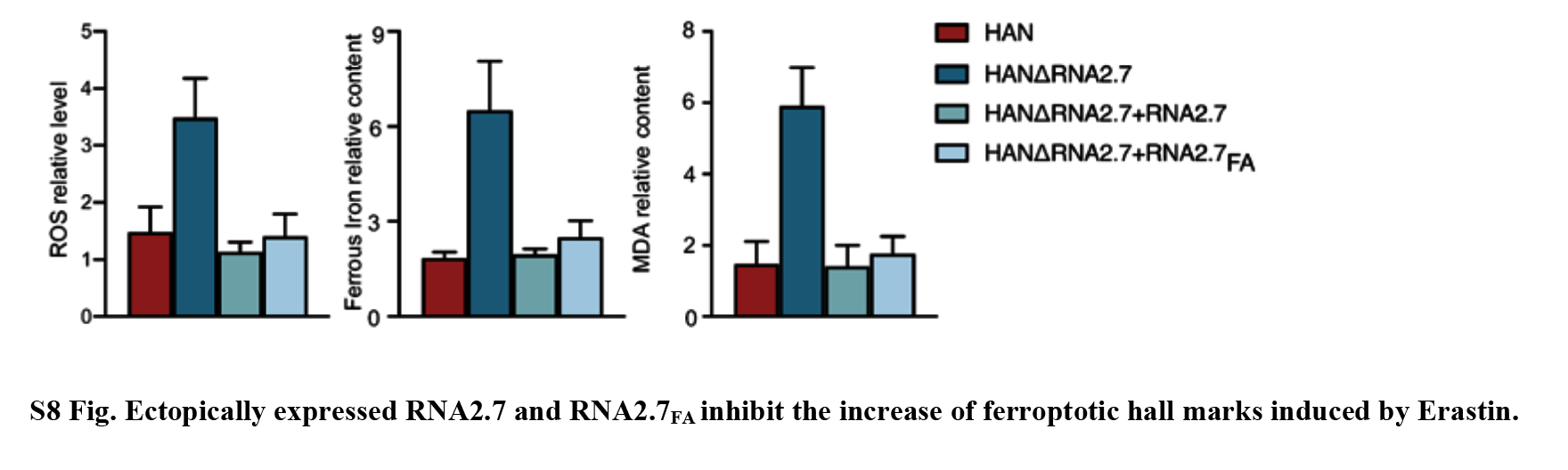

Supplement: S8 Fig — HELF cells were infected with HAN/HANΔRNA2.7 (MOI = 1). At 24 hpi, HANΔRNA2.7 infected HELF cells were transfected with vectors expressed RNA2.7/RNA2.7FA respectively. At 48hpi, Erastin was added into the supernatants with a final concentration of 5μM. Contents of reactive oxygen species (ROS), ferrous iron, and malondialdehyde (MDA) were measured using ROS assay, MDA assay and iron assay at 24 hours post treatment, respectively. Relative contents were calculated using mock group as control. All experiments were conducted independently three times. Error bars represent the mean ± SD from three independent experiments. (TIF) [file ppat.1012815.s012.tif]

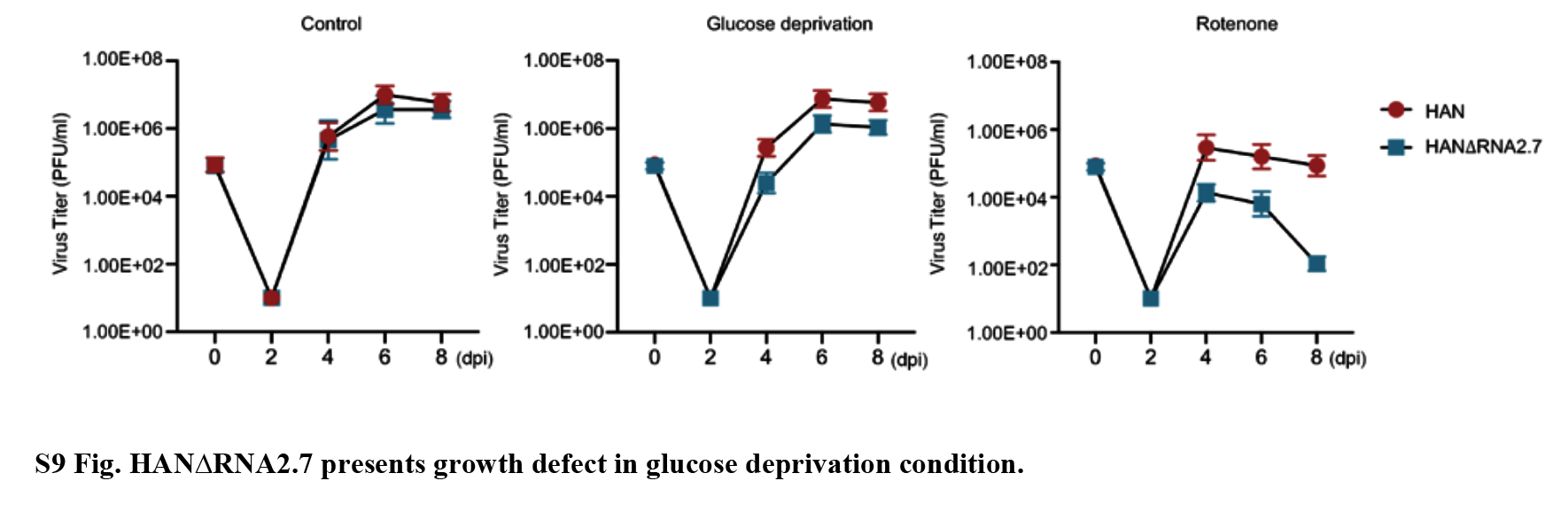

Supplement: S9 Fig — Growth of HAN, HANΔRNA2.7 in HELF cells under metabolically stressed condition. PFU, plaque-forming units. Cells were infected with HAN/HANΔRRNA2.7 at MOI = 0.1. At 24 hpi, HAN infected and HANΔR RNA2.7 infected HELF cells were incubated with normal media/ rotenone(5nM) or glucose-depleted media. The virus titer was performed by TCID50 assay and Error bars show mean±SD for three independent experiments. (TIF) [file ppat.1012815.s013.tif]

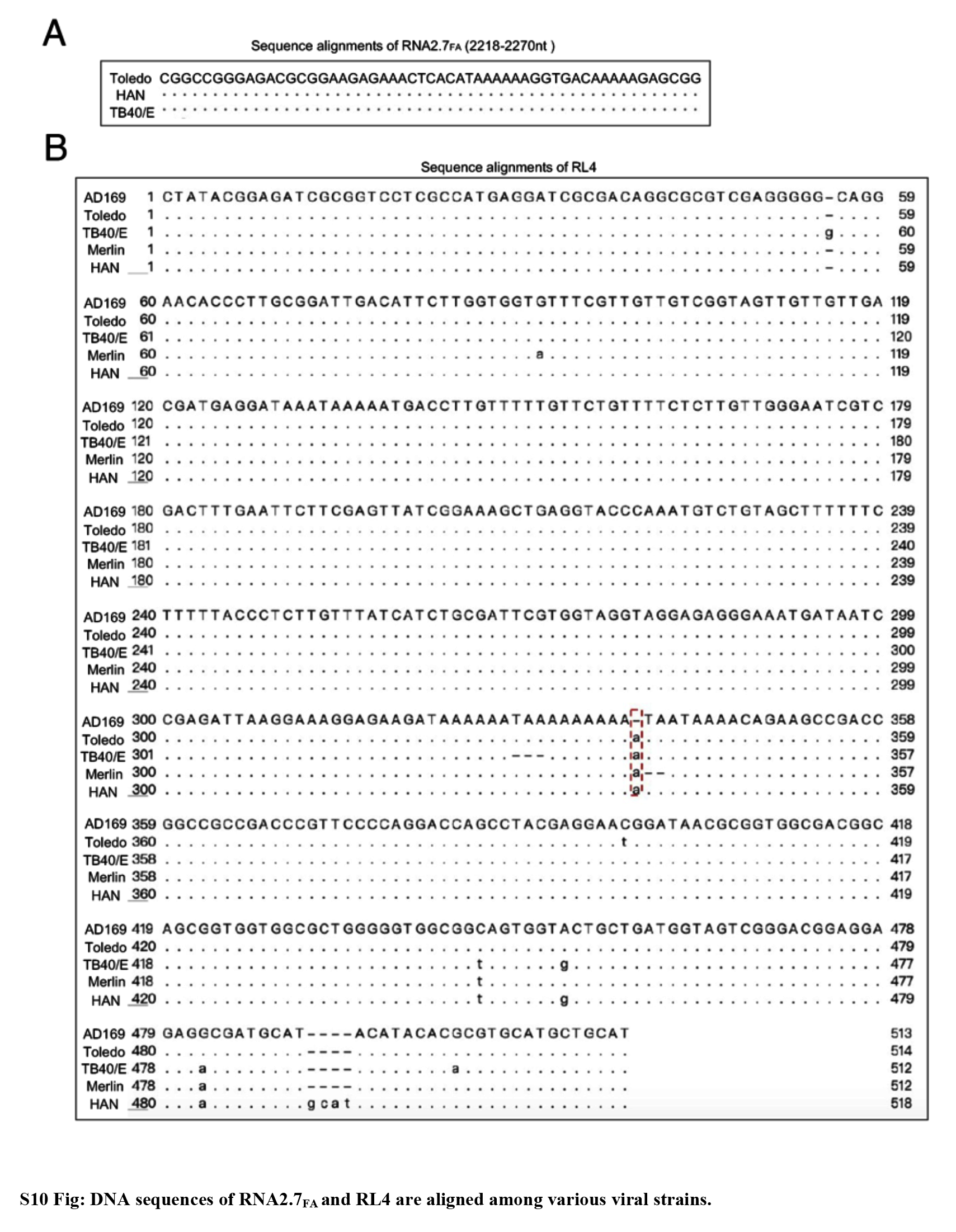

Supplement: S10 Fig — (A)The RNA2.7FA sequence of the Toledo strain was utilized as a reference, against which the sequences of other strains were compared. Dots indicate no differences from the reference sequence, while any mutant bases are shown accordingly. (B) The RL4 sequence of the AD169 strain was utilized as a reference, against which the sequences of other strains were compared. Dots indicate no differences from the reference sequence, while any mutant bases are shown accordingly. (TIF) [file ppat.1012815.s014.tif]
